# Disentangling Reaction Processes of Zeolites within Single‐Oriented Channels

**DOI:** 10.1002/anie.201916596

**Published:** 2020-03-02

**Authors:** Donglong Fu, Onno van der Heijden, Katarina Stanciakova, Joel E. Schmidt, Bert M. Weckhuysen

**Affiliations:** ^1^ Debye Institute for Nanomaterials Science Faculty of Science Utrecht University Universiteitsweg 99 3584 CG Utrecht The Netherlands

**Keywords:** methanol conversion, structure–reactivity relationships, thin films, zeolites, ZSM-5

## Abstract

Establishing structure–reactivity relationships for specific channel orientations of zeolites is vital to developing new, superior materials for various applications, including oil and gas conversion processes. Herein, a well‐defined model system was developed to build structure–reactivity relationships for specific zeolite‐channel orientations during various catalytic reaction processes, for example, the methanol‐ and ethanol‐to‐hydrocarbons (MTH and ETH) process as well as oligomerization reactions. The entrapped and effluent hydrocarbons from single‐oriented zeolite ZSM‐5 channels during the MTH process were monitored by using operando UV/Vis diffuse reflectance spectroscopy (DRS) and on‐line mass spectrometry (MS), respectively. The results reveal that the straight channels favor the formation of internal coke, promoting the aromatic cycle. Furthermore, the sinusoidal channels produce aromatics, (e.g., toluene) that further grow into larger polyaromatics (e.g., graphitic coke) leading to deactivation of the zeolites. This underscores the importance of careful engineering of materials to suppress coke formation and tune product distribution by rational control of the location of zeolite acid sites and crystallographic orientations.

## Graphical abstract

**A well‐defined model system** was developed to establish structure–reactivity relationships for specific zeolite‐channel orientations during catalytic reactions, such as the methanol‐to‐hydrocarbons process. Straight zeolite‐channels favor the formation of internal coke, promoting the aromatic cycle, and sinusoidal zeolite‐channels produce aromatics that eventually grow into condensed polyaromatics at the surface leading to deactivation of the zeolites.

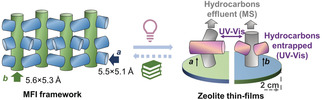

Understanding structure–reactivity relationships of heterogeneous catalysts is of utmost importance for rational improvement of their performance and lifetime.^1^, ^2^ Designing model systems with controlled active sites has been demonstrated to be a powerful approach for unravelling the structure–reactivity relationships in metal‐based catalysis. A well‐documented example is ammonia synthesis, for which surface science studies have revealed a lot of detailed insights into the working principles.^3^, ^4^ In microporous materials (MMs), it is difficult to create analogous model systems because the reactivity of these materials is not only determined by active sites, but also is heavily dictated by the pores and/or cavities as well as facets and even material defects.^5^, ^6^ Moreover, no robust methods are currently known to control the location of active sites in specific channels/pores for the study of working principles. Commercially, one of the most important MMs is zeolite ZSM‐5 (MFI framework topology) with two different types of channels, namely, sinusoidal and straight channels (Figure 1 a). This unique channel structure and resulting performance have made zeolite ZSM‐5 ubiquitous in petroleum refining and chemical manufacturing. However, the detrimental formation of coke limits catalyst activity and lifetime, serving as a major roadblock in the application of the zeolite materials in any high‐demand catalytic processes, such as the methanol‐to‐hydrocarbons (MTH) process.^7^, ^8^, ^9^, ^10^, ^11^, ^12^

**Figure 1:**
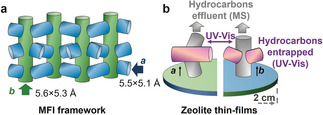
a) Micropore topology of the MFI zeolite framework type. Arrows indicate zeolite‐channels aligned in the *a*‐ and *b*‐directions. b) Schematic of operando UV/Vis diffuse reflectance spectroscopy (DRS) and on‐line mass spectrometry (MS) over *a*‐oriented and *b*‐oriented zeolite ZSM‐5 thin films for the establishment of structure–reactivity relationships for specific zeolite‐channel orientations.

ZSM‐5 coking in the MTH process has long been studied,^13^ and it is known that the deactivation is caused by the coking of aromatic species, and that the deactivation heavily depends on the location of the coke.^13^, ^19^, ^20^, ^21^, ^22^, ^23^, ^24^ To fully elucidate the key descriptor for zeolite deactivation, it is of utmost importance to study the reaction behaviors at the level of single‐oriented zeolite‐channels. Previous studies on zeolite ZSM‐5 in the MTH reaction have concentrated on either designing materials/reactors for kinetic studies^11^, ^16^, ^20^, ^22^, ^24^, ^25^ or gaining spatial insights by using advanced characterization techniques.^17^, ^18^, ^26^, ^27^, ^28^, ^29^, ^30^, ^31^ However, none of these studies have been capable of disentangling the structure–reactivity relationships for specific zeolite‐channel orientations under operando conditions.

Herein, as shown in Figure 1 b, uniformly oriented zeolite ZSM‐5 thin films were developed as well‐defined model systems to build the required structure–reactivity relationships at the level of single‐oriented zeolite‐channels during the MTH process. Highly *a*‐oriented and *b*‐oriented, catalytically active, zeolite ZSM‐5 thin films (Figures 2 b,d) were fabricated by using previously developed methods and grown from monolayers (Figures 2 a,c) with corresponding crystallographic orientations.^32^, ^33^, ^34^ The preferential orientations of the thin films were examined by X‐ray diffraction (XRD, Figure 2 e).^32^, ^33^, ^34^ Operando UV/Vis diffuse reflectance spectroscopy (DRS) coupled with on‐line MS was used to investigate the MTH process over the oriented zeolite ZSM‐5 thin films.^8^, ^35^, ^36^ The results (Figures 3 a,b and S3) reveal distinct UV/Vis absorption of hydrocarbon species in the two oriented thin films, illustrating different coking behaviors.

**Figure 2:**
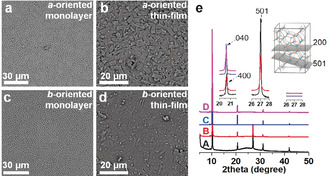
a,b) Scanning electron microscopy (SEM) images of a) an *a*‐oriented silicalite‐1 monolayer and b) the zeolite ZSM‐5 thin film grown from (a). c,d) SEM images of c) a *b*‐oriented silicalite‐1 monolayer and e) the zeolite ZSM‐5 thin film grown from (c). e) Traces (A), (B), (C), and (D) display representative X‐ray diffraction (XRD) patterns of the samples in (a), (b), (c), and (d), respectively. Note that the thin films in (b) and (d) are grown in the same secondary growth media solution with Si/Al=45.

**Figure 3:**
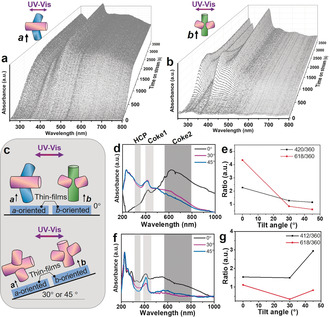
a,b) Operando UV/Vis diffuse reflectance spectra (DRS) of a) an *a*‐oriented and b) a *b*‐oriented zeolite ZSM‐5 thin film grown from a secondary growth media with Si/Al=45 during the methanol‐to‐hydrocarbons (MTH) process at 673 K. c) Schematic representation of the UV/Vis DRS study of the zeolite ZSM‐5 thin films in their tilted (0, 30, and 45°) positions. d,f) UV/Vis DRS spectra from the d) *a*‐oriented and f) *b*‐oriented thin film measured with corresponding tilted angles after 1.5 h MTH reaction. e,g) The UV/Vis DRS intensity ratios of the absorption bands at ≈420 nm (small coke species, coke1) and ≈620 nm (large coke species, coke2) to ≈360 nm (HCP species) in terms of different tilted angles of the thin films over e) *a*‐oriented and g) *b*‐oriented thin films, respectively.

The uniform crystallographic structure of oriented zeolite thin films allows us to precisely locate the positions of small aromatic cations (e.g., linear polyaromatics of naphthalene and anthracene, coke1) at ≈420 nm and polyaromatics (e.g., four/five‐ring aromatics, coke2) at >560 nm (see assignments in Table S3).^19^, ^22^, ^37^, ^38^ This was made possible by adjusting the relative angle between the electric field of the excitation light and the dipole moment of the coke species by tilting their positions (Figure 3 c). The maximum absorption is obtained when the electric field of the excitation light is parallel to the dipole moment of the entrapped molecules. Tilting the zeolite ZSM‐5 thin films changes their relative angle, thereby altering the absorption intensity (Figures 3 d,f). Hydrocarbon pool (HCP) species with an absorption at ≈360 nm were believed to be located in the channel intersections of MFI zeolites without preferential orientations, therefore, their dipole moment will be independent of the tilting angle of the thin films.^39^ Furthermore, tilting angle of the thin films alters the measurement area and reflectivity of the materials, thereby changing the absolute absorption intensity. Therefore, to exclude this effect, the intensity of the absorption at ≈360 nm was used to normalize the intensity of the absorption at ≈420 nm and above ≈560 nm. Figure 3 e and 3 g show a sharp decrease of the ratio of the ≈420 to ≈360 nm absorption bands when the *a*‐oriented thin film (Figure 3 e) was tilted from 0° (flat) to 45°, whereas an apparent increase was shown in its *b*‐oriented counterpart (Figure 3 g). This finding demonstrates that the coke species with an absorption at ≈420 nm are channel‐orientation dependent, and thus they are trapped inside the zeolite‐channels (internal coke). Conversely, as shown in Figure 3 e and 3 g, the change of the intensity ratio of the ≈620 to ≈360 nm absorption bands is independent of the channel orientations, and the ratios decreased in both zeolite thin films when they were tilted from 0 to 45°, suggesting that these coke species are located at the surface of the zeolite thin films (external coke).

After obtaining the precise location of different coke species, their evolution in specific zeolite‐channel orientations was analyzed from the thin films at the horizontal position. Figure 4 a shows a much higher intensity ratio of the absorption bands at ≈420 nm (naphthalene/anthracene carbocations) to ≈360 nm (cations of HCP species) in the straight channels than that in the sinusoidal channels. This result demonstrates that internal coke species can be readily formed in the straight channels.^29^ The observations are further supported by density functional theory (DFT) calculations.^39^ The results, summarized in Table 1 and Figures S4–S7, show that all molecules prefer to locate within the straight channels (and their intersections), particularly for anthracene with the largest energy difference of 86.9 kJ mol^−1^. Furthermore, severe distortion of large molecules, for example, naphthalene and anthracene, was observed in the sinusoidal channels, but not in the straight channels, indicating that there is extra strain in the planar conformation of these molecules. Therefore, the formation of naphthalene and anthracene will be impeded in the sinusoidal channels, but favored in the straight channels.^29^, ^39^ Additionally, because the largest molecule that can diffuse out of the channels with 10‐membered rings is 1,2,4‐trimethylbenzene, we believe that internal coke (once formed) cannot diffuse out from the 10‐membered‐ring channels, and its fast accumulation leads to pore blockage in the straight channels faster than in the sinusoidal channels. This process leads to the deficiency of methanol in the *b*‐oriented zeolite thin films, resulting in a fast consumption and disequilibrium of HCP species after 10 min (see details in the Supporting Information, Figure S8). Therefore, instead of using HCP species as a reference, we investigated the intensity ratio of external to internal coke species for the evaluation of coking behaviors at the surface of the zeolite thin films. The results (Figure 4 b) show a much higher intensity ratio of external to internal coke species at the surface of the sinusoidal channels for TOS >300 s (see detailed discussion in Figure S3). This result was further corroborated by using confocal fluorescence microscopy (CFM, excitation laser=560 nm, Figure S9), showing much stronger fluorescence emission at the wavelength >580 nm in the *a*‐oriented zeolite thin films.^17^ Additionally, similar UV/Vis DRS results in regards to internal and external coke species were also observed in zeolite thin films grown from a secondary growth media (SGM) solution with Si/Al=125 (Figure S10). Thus, the UV/Vis DRS results suggest that internal coke species can be readily formed in the straight channels, and the external coke species are more rapidly formed at the surface of the sinusoidal channels.

**Figure 4:**
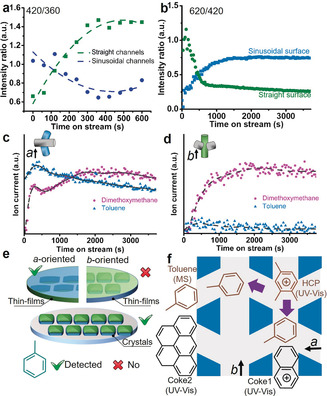
a) Temporal changes of the intensity ratio of internal coke species at ≈420 nm to hydrocarbon pool (HCP) species at 360 nm for the sinusoidal channels (blue) and the straight channels (green) during the methanol‐to‐hydrocarbons (MTH) process at 673 K as a function of time on stream (TOS) from 0 to 600 s. b) Temporal changes of the intensity ratio of external coke species at ≈620 nm to internal coke species at ≈420 nm for *a*‐oriented thin films (blue) and *b*‐oriented thin films (green) during the MTH process at 673 K as a function of TOS from 0 to 4000 s. c,d) On‐line mass spectrometry (MS) profiles of dimethoxymethane and toluene over c) an *a*‐oriented and d) a *b*‐oriented thin film with Si/Al=45. e) Schematic of toluene effluent from *a*‐oriented (blue) and *b*‐oriented (green) zeolite ZSM‐5 thin films, as well as discrete zeolite ZSM‐5 crystals. f) Schematic illustrations of the location of coke species and the aromatic formation determined by UV/Vis or MS results for the MTH process. Dashed lines in (c) and (d) are interpolations to guide the eyes.

**Table 1:** The calculated adsorption energies of different hydrocarbon molecules in silicalite‐1.^[a]^

Molecules		Adsorption energy intersection [kJ mol^−1^]			Adsorption energy channels [kJ mol^−1^]		
		Sin.	Str.	Δ*E*_b−a_	Sin.	Str.	Δ*E*_b−a_
Product	toluene	−86.5	−89.7	3.2	−59.2	−71.8	12.6
Coke [420 nm]	naphthalene	−88.2	−92.6	4.4	−44.2	−67.4	23.2
	anthracene	−99.8	−111.1	11.3	12.1	−74.8	86.9

[a] Sin. and Str. represent sinusoidal and straight channels, respectively.

Simultaneously to the UV/Vis DRS measurement, the reaction products (Figure 1 b) emanating from the sinusoidal and straight zeolite ZSM‐5 channels were monitored from the *a*‐oriented and *b*‐oriented thin films, respectively. Representative on‐line MS spectra (Figures 4 c,d and S11) show toluene (*m*/*z=*91) in the effluent products from the sinusoidal channels, whereas this molecule is absent (or in trace amount) in the emanating products from the straight channels. ^13^C‐Labeled MTH experiments further confirmed the appearance of toluene from the sinusoidal channels with an MS peak at *m*/*z=*98 (Figures S11 and S12). Similar results (Figure S13) were also observed in the thin films grown in the SGM with Si/Al=125. Interestingly, discrete *b*‐oriented zeolite ZSM‐5 crystals (Figure 4 e), with the sinusoidal channels being accessible, show a strong toluene peak at 98 (^13^C methanol, Figure S12), further corroborating that toluene preferentially diffused out from the sinusoidal channels. Toluene is formed in the zeolite channel intersections and only small energy differences between the two channel orientations were found (Table 1), therefore, the differences in the emanating products are due to further oligomerization reactions of toluene in the straight channels.^40^ As shown in Figure 4 f, derived from the MS results, UV/Vis DRS data, and DFT calculations, we have evidence that the straight zeolite ZSM‐5 channels are quickly blocked, preventing the production of toluene.^31^ In the constrained sinusoidal channels the internal coke formation was suppressed, allowing toluene to easily diffuse from these zeolite channels and further convert into polyaromatics (after successive alkylation) on the corresponding surface.^31^

To determine the generality of our model system for catalysis research, three other zeolite‐catalyzed reactions (Figure S14–16), namely 4‐methoxystyrene and thiophene oligomerization as well as the ethanol‐to‐hydrocarbons (ETH) process, were tested. The UV/Vis DRS results (Figures S14 and S15) suggest that larger products or linear polyaromatic coke molecules in all three reactions can be readily formed within the straight channels. Moreover, toluene also preferentially diffused out from the sinusoidal channels during the ETH process, as shown in Figure S16. It should be noted here that the maximum ion current of dimethoxymethane (DMM) was detected (Figures 4 c and S13 a) earlier than that of toluene during the MTH process. In contrast, the maximum ion current of DMM was detected (Figure S16 a) after that of toluene during the ETH process. This distinct observation is because DMM is related to the first C−C formation (Figure S13 c), which is unnecessary in the ETH process.^41^ Thus, these experiments also suggest that the thin films can be used as catalytic model systems to study chemical reaction mechanisms.

Finally, to further disentangle the key descriptor for the deactivation of zeolite ZSM‐5 catalysts, anisotropic zeolite ZSM‐5 crystals with different channel orientations dominating the surface, as well as comparable physicochemical properties (Figures 5 a,b and S17, Table S4), were prepared. For both the same weight hourly space velocity (WHSV, Figure 5 e) and similar starting conversion (Figure S18 a) of methanol, remarkable differences in the deactivation behaviors for the two distinct catalyst systems were obtained. The results show a constant and high deactivation rate for the *a*‐oriented catalysts, and two distinct deactivation stages for the *b*‐oriented catalysts. The simultaneous measurement of UV/Vis DRS (Figures S18 b and S18 c) at the same WHSV showed similar starting spectra, suggesting comparable starting intermediates in the two materials.^8^ Interestingly, as shown in Table S5, a high intensity ratio (≈1.0) of ≈640 to ≈420 nm at both 10 and 48 h was observed for the *a*‐oriented crystals, demonstrating a heavy external coke deposition at the surface. This finding is consistent with the conclusions from the thin films. However, an apparent increase of the intensity ratio for the *b*‐oriented crystals was observed from 0.81 at 10 h to 0.99 at 48 h, suggesting the gradual accumulation of external coke species. The thermogravimetric analysis (TGA) MS results (Figure S19) revealed more coke species in the *a*‐oriented zeolite crystals than in the *b*‐oriented zeolite crystals, consistent with the faster deactivation of the *a*‐oriented zeolite crystals. These observations clearly suggest that external coke formation dictates the zeolite‐catalyst deactivation. Furthermore, Figure 5 e showed a higher ethylene/2MB (2‐methylbutene and 2‐methylbutane) ratio for the *b*‐oriented catalysts. The ethylene/2MB ratio is often used as a descriptor assessing the propagation of the aromatic‐ and olefin‐based cycles of the HCP mechanism (Figure 5 c).^42^ Therefore, the prompt coke formation in the straight channels enhanced the aromatic cycle in the *b*‐oriented zeolite ZSM‐5 crystals with a high ethylene/2MB ratio.

**Figure 5:**
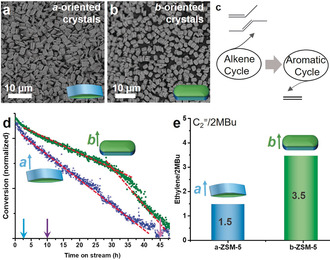
a,b) Scanning electron microscopy (SEM) images of the a) *a*‐oriented and b) *b*‐oriented zeolite ZSM‐5 crystals with Si/Al=125 in the synthesis solution. c) Simplified dual‐cycle hydrocarbon pool (HCP) mechanism of the methanol‐to‐hydrocarbons (MTH) process. d) Deactivation profiles of the anisotropic zeolite ZSM‐5 crystals during 48 h of the MTH process at 623 K with an identical WHSV of 5 h^−1^. e) The ratio of ethylene to 2‐methylbutene and 2‐methylbutane (2MBu) for the anisotropic zeolite ZSM‐5 crystals.

Summarizing, the present work developed a model system, i.e., uniformly oriented zeolite thin films, for the establishment of the structure–reactivity relationships of zeolite‐catalyzed reaction processes at the level of single‐oriented channels. The entrapped and effluent hydrocarbons from a single‐oriented zeolite‐channel were monitored by using operando UV/Vis DRS and on‐line MS, respectively. Investigation of the methanol‐to‐hydrocarbons (MTH) process over zeolite ZSM‐5 demonstrated that the straight channels favor the formation of internal coke, promoting the aromatic cycle. It also showed that the sinusoidal channels produce aromatics (e.g., toluene) that further grow into larger polyaromatics at the surface (e.g., graphitic coke) leading to deactivation of the zeolites. This study highlights the importance of controlling the location of zeolite acid site as well as preferential crystallographic orientations to suppress coke formation and tune product distribution. The successful application of the developed model system also offers a new approach to study zeolite chemistry at the level of single‐oriented zeolite‐channels by rational design of model systems.

## Conflict of interest

The authors declare no conflict of interest.

## Supporting information

As a service to our authors and readers, this journal provides supporting information supplied by the authors. Such materials are peer reviewed and may be re‐organized for online delivery, but are not copy‐edited or typeset. Technical support issues arising from supporting information (other than missing files) should be addressed to the authors.

Supplementary
