# Supplementary material for: Disentangling Reaction Processes of Zeolites within Single‐Oriented Channels
Source: Angew Chem Int Ed Engl. 2020 Mar 2;59(36):15502–6. doi: 10.1002/anie.201916596 (PMC7496746; doi:10.1002/anie.201916596)
Supplement: Supplementary file 1 — Supplementary [file ANIE-59-15502-s001.pdf]

Angewandte Chemie
Eine Zeitschrift der Gesellschaft Deutscher Chemiker

Supporting Information

# Disentangling Reaction Processes of Zeolites within Single-Oriented Channels

*Donglong Fu, Onno van der Heijden, Katarina Stanciakova, Joel E. Schmidt, and Bert M. Weckhuysen**

anie_201916596_sm_miscellaneous_information.pdf

# SUPPORTING INFORMATION

**Table of contents**

**1. Section S1: Experimental Details on Materials Synthesis…………………………………………S2**

**2. Section S2: Computational Methods……………………………………..…………...…………..S5**

**3. Section S3: Characterization………………………………………………………………………S7**

**4. Section S4: Supplementary Information Figures S1-S19 and Tables S1-S5………………..….S9**

**5. References……………………………………………………………………………………...S30**

## Section S1: Experimental Details on Materials Synthesis

### A. Chemical Materials

Tetraethyl orthosilicate (TEOS, 98% (GC), Sigma Aldrich), tetrapropylammonium hydroxide (TPAOH, 1M aq., Alfa Aesar), bis(hexamethylene)triamine, 1-iodepropane (99%, Sigma Aldrich), 2-butanone, anhydrous potassium carbonate (≥99.0% (T), anhydrous, BioUltra, Sigma-Aldrich), diethyl ether (ACS reagent, , ≥99.0%, anhydrous, ACS reagent, Sigma-Aldrich), ethyl acetate (99.8%, anhydrous, Sigma-Aldrich), potassium hydroxide (KOH, pellets, 85%, Alfa Aesar), aluminium sulphate hexadecahydrate ($Al_2(SO_4)_3 \cdot 18\ H_2O$, 98%, Sigma-Aldrich), ammonium nitrate ($NH_4NO_3$, >99%, Acros Organics), sodium silicate solution ($Na_2O(SiO_2)_x \cdot xH_2O$, ~10.6% $Na_2O$, ~26.5%$SiO_2$, Sigma-Aldrich), ethanol (anhydrous, absolute, Biosolve), 4-methoxystyrene (97%, Sigma-Aldrich), thiophene (GC standard, Sigma-Aldrich), methanol (99.8 %, Sigma-Aldrich), sulfuric acid (95%, reagent grade, Fischer scientific), quartz plates (LSP Quartz B.V., 20 mm in diameter), glass plates (VWR, 22 mm in diameter), branched polyethylenimine (PEI, Mw= 25000, Mn 10000, Sigma-Aldrich) were used as received.

### B. Synthesis of *a*-Oriented and *b*-Oriented Slicalite-1 Crystals and Zeolite ZSM-5 Crystals

a. Structure-directing agents (SDAs) for the crystals

Trimer-tetrapropylammonium cation (trimer-$TPA^{3+}$, Figure S1) was reported as the structure-directing agents (SDAs) for *a*-oriented crystals. The synthesis of trimer-$TPA^{3+}$ was adapted from Tsapatsis's method,[1] where bis(hexamethylene)triamine was alkylated with 1-iodopropane, as shown in Figure S1. Typically, 450 mL of the solvent 2-butanone, an excess of 72.6 g of anhydrous potassium carbonate as HI and water scavenger, and 27.87 g of bis(hexamethylene)triamine were added to a three neck 1 L round bottom flask. A dropping funnel and a reflux condenser were connected to the flask. The reaction flask was located in a silicone oil bath on a stirring plate, flushed with nitrogen gas, and vented from the top of the condenser. The whole set-up was wrapped by aluminum foil to avoid iodide oxidation, which is sensitive to light. The solution was gently heated and refluxed at 353 K under nitrogen atmosphere. Then 101 mL of 1-iodopropane was added dropwise with an addition funnel. The reaction was conducted overnight (~ 15 h) followed by filtration to remove the 2-butanone. The recovered solids contain the target product, trimer-$TPA^{3+}3I^{-}$ along with impurities, *i.e.,* KI and $K_2CO_3$. The solids were dissolved in 100 ml ethanol for several h followed by filtration to obtain the filtrate. An off-white solid, trimer-$TPA^{3+}3I^{-}$ with a small amount of impurity, was obtained by removing ethanol by rotary evaporation. The purification was done by adding in 250 mL of cold 2-butanone, after which equal volume of ethyl acetate was slowly added to precipitate out trimer-$TPA^{3+}3I^{-}$. After 10 h stirring the target product was recovered from the solution by filtration. A higher purity of the target product can be obtained by repeating the latter part, *i.e.,* the cycle of ethanol extraction and recovery by diethyl ether and ethyl acetate. The purity was checked by $^{13}C$ NMR (Figure S1). TPAOH was used as received for the synthesis of *b*-oriented zeolite crystals.

b. Preparation of *a*-oriented and *b*-oriented silicalite-1 crystals

For the synthesis of the *a*-oriented silicalite-1crystals with a leaf-like shape, a solution with a ratio of 6 TEOS: 0.75 timer-$TPA^{3+}$: 3.75 KOH: 1425 $H_2O$ was prepared. After stirring for 8 h. the solution was poured into an autoclave through a filtration paper. The autoclave was placed in a rotational oven at 448 K for 24 h. The preparation of *b*-oriented silicalite-1 crystals followed Yoon's method.[2] The round, silicalite-1 crystals were synthesized from a solution composition of 6 TEOS:0.9 TPAOH:620 $H_2O$. The synthesis solution was prepared by adding TEOS to the solution containing TPAOH and $H_2O$. The mixture was transformed into a clear solution after stirring in a sealed liner for 24 h at room temperature. The clear solution was filtered with filter paper and charged into a Teflon-lined autoclave. The hydrothermal reaction was carried out under rotation oven at 423 K for 12 h.

c. Preparation of *a*-oriented and *b*-oriented zeolite ZSM-5 crystals

The *a*-oriented zeolite ZSM-5 crystals were prepared with the method adjusted from literatures.[1,3] Typically, the synthesis solution of *a*-oriented ZSM-5 crystals was prepared by introducing TEOS into the solution containing trimer-$TPA^{3+}$, KOH and $H_2O$. Then the required amount of $Al_2(SO_4)_3{\cdot}18\ H_2O$ was added to the clear mixture (pH≈ 13) obtained after stirring in a sealed liner for 4 h at room temperature. The final solution with a composition of 6 TEOS:0.024 Al:0.75 rimer-$TPA^{3+}$:1.17 KOH:620 $H_2O$ was obtained after stirring at room temperature for 1 extra hour. This solution was subsequently poured into a Teflon liner. This liner was placed in an autoclave and heated to synthesis temperature (448 K) in a rotation oven for 24 h. Preparation of *b*-oriented zeolite ZSM-5 crystals was adapted from an earlier reported method.[2] In a typical synthesis, the synthesis solution was prepared by adding TEOS to the solution containing TPAOH and $H_2O$, that was stirred for 4 h to allow for the hydrolysis of TEOS. Then required amount of $Al_2(SO_4)_3{\cdot}18\ H_2O$ was added to this solution with a final composition of 6 TEOS:0.024 Al:0.9 TPAOH:620 $H_2O$. The solution was transferred into 20 mL Teflon-lined stainless steel autoclaves, and the hydrothermal reaction was carried out under rotation oven at 443 K for 24 h.

All the crystals were calcined in an air oven at 823 K for 6 h with a ramp rate of 1.0 K/min to remove $TPA^+$ or timer-$TPA^{3+}$. These samples were converted to an acid form (Brønsted acids) by $NH_4^+$-ion exchange of the calcined zeolites with 1.0 M ammonium nitrate solution at 353 K overnight followed by calcination for 6 h at 823 K with a ramp rate of 1.5 K/min.

**C. Synthesis of Uniformly *a*-Oriented and *b*-Oriented Thin films**

a. Pretreatment of the substrate

The quartz plates were first immersed into $H_2O_2$ (30 wt% aqueous solution) for at least 30 min to remove the surface organics. Then the substrates were rinsed with deionized $H_2O$ and dried at 333 K. The $H_2O_2$ pretreated quartz plates were directly used for *b*-oriented monolayer deposition. For the fabrication of *a*-oriented monolayers, the organic free quartz plates were placed into a 0.425 wt.% PEI solution (ethanol) for 2 min followed by drying at 333 K under static air in a conventional oven for 20 min. This procedure was repeated for at least three times.

b. Manual assembly of an *a*-oriented and a *b*-oriented silicalite-1 monolayer

Typically, *ca*. 30 mg of *a*-oriented and *b*-oriented silicalite-1 crystals were put onto the surface of the substrates followed by pressing and rubbing using a finger. To avoid any contamination of the substrates surface and zeolite crystals with moisture or other pollutants from the finger, a clean soft latex glove was placed tightly on the finger. Subsequently, the loosely attached top layers were removed by gently wiping using glass wool for about 30 s. The *a*-oriented monolayers were calcined at 823 K overnight to remove the organic layers before secondary growth. The *b*-oriented monolayers were calcined for 2 h at 423 K to enhance the strength of the attachment.

c. Preparation of *a*-oriented and *b*-oriented zeolite ZSM-5 thin films on quartz plates

The silicalite-1 monolayers supported on quartz plates were intergrown by the secondary growth method (SGM) at 448 K for 24 h. Typically, a solution comprising a required amount of $Al_2(SO_4)_3 \cdot 18\ H_2O$, 0.77 g $H_2SO_4$ (10 wt% aqueous solution) and 6.9 g of $H_2O$ was added to a sodium silicate solution made by mixing 0.977 g of sodium silicate and 7.31 g $H_2O$ as well as 0.32 g ethanol. After another 30 min of stirring at room temperature, a clear solution (Si/Al= 125 or 45) was obtained and was transferred without filtration to a 20 mL Teflon-lined autoclave with the *a*-oriented and *b*-oriented crystals seeded substrates vertically placed at the bottom of the same autoclave. After the reaction, the autoclave was removed from the oven and quickly cooled to room temperature by immersing in cool $H_2O$. As-synthesized zeolite membranes on the substrates were carefully removed from the autoclave and washed with copious amounts of deionized $H_2O$ and dried in air at 333 K.

After the synthesis, the thin films were first treated with 0.2 M $NH_4F$ solution for 2 h without stirring to remove the outermost amorphous silica layers that can block the channels of the membranes.[2] Then the thin films were calcined at 823 K overnight with a ramp rate of 1.5 K/min under an air flow to remove the organic additive, *i.e.,* ethanol. The H-form sample was obtained by three times repeating the ion exchange of the calcined Na-type sample with a 1 M $NH_4NO_3$ solution (Acros Organic, 99+%) at 333 K overnight followed by a calcination at 823 K for 6 h at 823 K with a ramp rate of 1.5 K/min.

## Section S2: Computational Methods

Periodic density functional theory (DFT) calculations based on the approach of mixed Gaussian and Plane Waves (GPW)[4] and Perdew–Burke–Ernzerhof (PBE) functional were carried out using the CP2K Software.[4,5] To account for dispersion forces the correction scheme of Grimme was added.[6] As shown by Gölt *et al.*, PBE+d functional correctly reproduces adsorption energies with an average error of *ca.* 5 kJ/mol in the study of the adsorption of hydrocarbons in zeolites.[7,8] For all simulations we have used GTH pseudopotentials,[9] TZVP basis[10] set and a plane wave cutoff 700 Ry. The accuracy of SCF cycle was set to $10^{-7}$ charge units. Purely siliceous MFI, with an orthorhombic unit cell was studied. Although silicalite-1 is inactive for MTH process, we used it as a model system to study the stability of various HCP species inside zeolite channels. The interaction of HCP species with a zeolite has two contributions: weak (and long-range) interactions between the adsorbate and zeolite walls and short-range directional interactions with an acid site. As has been shown by Brogaard *et al*., the weak van der Waals interactions are the key factor that determines the stability of arenes in the zeolite ZSM-5 and even in the presence of easily accessible acid sites in the sinusoidal and straight channels is the adsorption in an intersection region without an acid site still preferred.[11] Similar results obtained Boronat and Corma who found that the stability of small probe molecules (such as pyridine) and their observed acidity, is rather determined by confinement effect than by differences in the acid strength of Al sites.[12] Moreover, the *a*-oriented and *b*-oriented zeolite ZSM-5 thin films in the present work are grown from the same secondary growth media solutions. Therefore, the influence of Al sites on the stability of adsorbates over the zeolite will be on average everywhere the same and the introduction of Al sites into our model would not qualitatively change our results.

Initially, to avoid the extra strain, the experimentally observed lattice parameters (*a*= 20.090 Å, *b*= 19.738 Å and *c*= 13.142 Å) were optimized by relaxing the unit cell leading to the new lattice parameters (*a*= 20.182 Å , *b*= 19.823 Å and *c*= 13.263 Å ). These values were then used in all subsequent calculations. The potential energy profiles along the zeolite ZSM-5 channels were explored using Nudged Elastic Band (NEB) method by calculating 20 different locations in the corresponding channels, shown in Figure S4. NEB is a method for finding saddle points and minimum energy paths (MEP) between pre-defined end points. To ensure that the true saddle point along the MEP has been found, we refined the NEB results by climbing image nudged elastic band calculations (CI-NEB).[13] The stable endpoints for subsequent NEB calculations were localized by a series of geometry optimizations of coke precursors (toluene, naphthalene and anthracene) with different locations and orientations along the channels of silicalite-1. The most stable structures were considered as starting points for NEB. For the calculations along the straight channels, the endpoints were localized in the intersection of the sinusoidal and straight channel with the orientation of molecules along the straight channel. A single cell contains two intersections along one channel, therefore 2 NEB calculations with a total of 40 locations were used to explore the potential energy along the straight channels. Because the topology along the sinusoidal channels is more complicated, NEB calculations between intersections were divided in two subsequent calculations with the intermediate point being the coke precursor localized in the sinusoidal channel. Thus, 4 NEB calculations were performed with a total of 80 images. To ensure that the results are independent from the direction of pulling the reverse NEB were performed

on the calculated molecules, the extent of confinement imposed on the molecules was expressed via a coordination number. The coordination number CN of molecules was computed using the following definition[14]:

$$CN = \sum_{C_{AB}} C_{AB}$$

where $C_{AB}$ is the coordination number between two groups A and B defined as:

$$C_{AB} = \sum_{i=1}^{N_A} \frac{1}{N_A} \left[ \sum_{j=1}^{N_B} \frac{1-(\frac{r_{ij}}{d_{AB}})^6}{1-(\frac{r_{ij}}{d_{AB}})^{12}} \right]$$

where $N_A$ and $N_B$ are the numbers of atoms of species A and B, $r_{ij}$ are the interatomic distances and $d_{AB}$ are scale parameters, which values were chosen as a sum of the Van der Waals radii for the given combination of atoms, thus: $d_{SiH=}$ 2.99 Å, $d_{OH=}$ 2.48, $d_{SiC=}$ 3.46 Å and $d_{SiC=}$ 2.95 Å.[15] The sigmoidal curvature of the function $C_{AB}(r_{ij})$ ensured that at $r_{ij} < d_{ij}$ the $CN \sim$ 1 meaning that there are steric constraints imposed on atom A from the atom B, while at distances at $r_{ij} > d_{ij}$, CN ~ 0 and there is no interaction between atoms A and B. The coordination number is a measure of the average number of Si and O framework atoms in the vicinity to every atom of the host molecule. It is computed as a summation over different Si-C, Si-H, O-H and O-C pairs and no intramolecular coordination is taken into the account (*i.e.,* the coordination of H atom to the C atom is omitted). The CN was moreover normalized with respect to the number of atoms in the given molecule.

## Section S3: Characterization

### A. Scanning Electron Microscopy

The images of the seed layers and thin films were examined using a scanning electron microscopy (SEM) from Phenome instrument operating at an accelerating voltage of 5 kV.

### B. X-ray Diffraction

X-ray diffraction (XRD) was used to confirm the orientation of the as-synthesized zeolite monolayers and thin films as well as membranes. Diffraction patterns were collected using a Bruker D2 Phaser (2$^{nd}$ Gen) instrument using a cobalt radiation source, Co $k_\alpha$= 1.789 Å. The samples were rotated at 15 revolutions/min.

### C. Temperature-Programmed Desorption of Ammonia

Temperature-programmed desorption of ammonia ($NH_3$-TPD) was performed on a Micromeritics Autochem II 2920 equipped with a TCD detector. Prior to TPD, *ca.* 100 mg of catalyst was first out gassed in He for 1 h at 873 K with a heating ramp of 10 K/min. Ammonia was adsorbed at 373 K until saturated, followed by flushing with He for 120 min at 373 K. The ammonia desorption was monitored using the TCD detector until 873 K with a ramp rate of 10 K/min, using a He flow of 25 mL/min.

### D. *Operando* UV/Vis Spectroscopy with On-Line Mass Spectrometry

The hydrocarbon pool species and related coke compounds formed during the methanol-to-hydrocarbons (MTH) processes were determined using *operando* UV/Vis diffuse-reflectance spectroscopy (DRS) coupled with on-line mass spectrometry (MS).[16,17] *Operando* UV/Vis DR spectra were obtained using an AvaSpec 2048L spectrometer connected to a high-temperature UV/Vis DRS optical fiber probe, which was used to collect spectra in reflection mode. The measurements were performed in the wavelength range of 200-1000 nm (11000-50000 cm$^{-1}$ in wavenumber).

Prior to the MTH process, two pieces of H-form *a*-oriented or *b*-oriented zeolite ZSM-5 thin films were placed on the heating stage of a Linkam cell, which was then heated to 623 K at a rate of 30 K/min and held at this temperature for 30 min in an $N_2$ atmosphere. Subsequently, acting as the carrier gas, a constant $N_2$ flow (15 mL/min) was introduced to a methanol saturator. At the same time, the UV/Vis DRS diffuse reflectance spectra and mass profiles were recorded simultaneously at a 30 s interval time for ~ 5800 s. At the end, the MTH process was quenched by rapid cooling of the Linkam cell by using Linkam TMS94 temperature controller. The mass spectrometry database from the National Institute of Standards and Technology (NIST) was consulted for referencing purposes. More specifically, the evolution of dimethoxymethane (DMM, m/z= 75), toluene (m/z= 91) and xylene (m/z= 106) were measured by monitoring the single mass signals. For $^{13}$C labelled reaction, DMM, toluene and xylene were monitored at m/z of 78, 98 and 117, respectively. Ethylene, propylene and dimethyl ether (DME) during $^{13}$C labelled reaction were also followed at m/z of 29, 44 and 47, respectively. The MS profiles of DMM and toluene were fitted using either Fourier 8 or polynomial fitting in Origin 9.0 to add interpolating lines for guiding the eyes.

**E. Fluorescence Micro-(Spectro)scopy**

Fluorescence micro-(spectro)scopy (FMS) was applied to visualize the coke species on zeolite thin films. Imaging was performed using a confocal fluorescence Nikon Eclipse 90i microscope equipped with a pin hole to filter out-of-focus light, and dichroic mirrors corresponding to the relevant laser line wavelength were used. Fluorescence microphotographs were collected using 561 nm (a 575 nm long-pass filter) laser light with a Nikon × 100/0.30 objective. The emission was detected at 575-720 nm (resolution of 10 nm) using an A1R scanning head equipped with a spectral detection unit consisting of a diffraction grating and a 32-photomultiplier tube array.

**F. Catalytic Testing of Anisotropic Zeolite ZSM-5 Crystals**

The catalytic testing of anisotropic zeolite ZSM-5 crystals was performed at 623 K in a quartz, rectangular fixed-bed reactor (ID= 6 mm× 3 mm). Typically, *ca.* 100 mg of catalysts was loaded with a weight hourly space velocity (WHSV) of 5 $h^{-1}$. A sieve fraction of 0.2-0.4 mm zeolite particles was used, and the resulting bed length was *ca.* 8 mm. A He flow with a methanol saturation of *ca.* 14.5 % was obtained by flowing the carrier gas through a saturator containing methanol at 293 K. *Operando* UV/Vis DR spectra were obtained using the same spectrometer and optical fiber probe as those in the testing of zeolite thin films. On-Line analysis of the reactant and reaction products was performed using an Interscience Compact (gas chromatography) GC, equipped with a Rtx-wax and Rtx-1 column in series and a Rtx-1, Rt-TCEP and $Al_2O_3$/$Na_2SO_4$ in series, both connected to an FID detector.

From the GC data obtained, the conversion was calculated as follows:

$$Conversion\ (\%) = \frac{[MeOH_{in}]-[MeOH_{out}]}{[MeOH_{in}]}$$

**G. Characterization of the Retained Hydrocarbons**

Thermogravimetric analysis-mass spectrometry (TGA-MS). To quantify the amount of coke formed during methanol conversion, thermogravimetric analysis (TGA) was performed with a Perkin Elmer Pyris 1 TGA thermogravimetric analyzer coupled to a Pfeiffer Vacuum Omnistar mass spectrometry (MS). To burn off the carbonaceous deposits, *ca.* 10 mg of spent catalysts were heated in pure $O_2$ flow to 1173 K with a ramp rate of 5 K/min, after drying in an Ar flow for 1 h at 393 K. The MS detector was set to monitor the evolution of $CO_2$ (m/z= 44).

## Section S4: Supplementary Information Figures S1-S19 and Table S1-S5

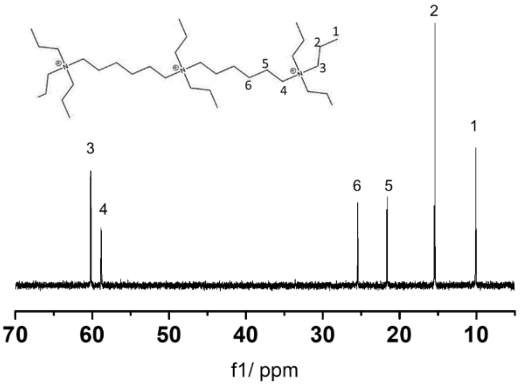


**Figure S1.** Nuclear magnetic resonance (NMR) spectrum of trimer-tetrapropylammonium cation (trimer-$TPA^{3+}$). The trimer-$TPA^{3+}$ has been used as the structure-directing agent for the synthesis of *a*-oriented purely siliceous MFI seeds and *a*-oriented zeolite ZSM-5 crystals.

**Preparation of an *a*-oriented monolayer on a glass/quartz plate**

**Table S1.** Synthesis approaches of *a*-oriented monolayer reported in literature.

| Methods | Monolayer attachment | Substrates | References |
|---|---|---|---|
| Spin coating of PEI | Manual assembly | PDMS modified Glass | Pham *et al.*[2] |
| Chemical deposition | Reflux | 3CP-TMS modified silica-coated α-$Al_2O_3$ | Choi *et. al*.[1,3] |
| Physical deposition | Sonification | α-$Al_2O_3$ | Kim *et al.*[18] |

**Table S2.** Methods of coating poly-ethylenimine (PEI) on top of a glass/quartz substrates[a]

| Method | | PEI (wt.%)[b] | Time (s) | Results |
|---|---|---|---|---|
| Spin coating/ rpm | 1500 | 0.5 | 15 | Poor coverage |
| | 2500 | 0.3 | 15 | Poor coverage |
| | 2500 | 0.5 | 15 | Fully covered, not densely packed |
| | 2500 | 1 | 15 | Fully covered, not densely packed |
| | 3500 | 0.5 | 15 | Fully covered, not densely packed |
| Dip coating | | 0.3 | 120 | Poor coverage |
| | | 0.425 | 120 | Best coverage, densely packed |
| | | 0.5 | 120 | Good coverage, densely packed |
| | | 0.75 | 60 | Fully covered, not densely packed |
| | | 1 | 60 | Fully covered, not densely packed |
| Drop coating[c] | | 0.425 | - | Best coverage, densely packed |

Note:

a. All PEI-coated substrates are heated for 20 min at 333 K and fully cooling down to room temperature before monolayer attachment.
b. PEI is dissolved into ethanol before PEI coating.
c. 2 droplets are dropped at the surface of a substrate for natural evaporation, which needs to be repeated for 3 to 6 times.
d. The highlighted boxes are the conditions that succeed for the continuous monolayer attachment.

As shown in Table S1, spin-coating followed by manual assembly as well as chemical functionalization of the substrate followed by refluxing/sonication were reported for the fabrication of *a*-oriented monolayers on glass or α-$Al_2O_3$ substrates, respectively.[1–3] In the present work, quartz plates were used as the substrates to prevent possible contaminations from glass plates. Due to the similar surface properties of quartz and glass plates, we first tested spin-coating of PEI on plates. However, monolayer with sparsely distributed crystals formed on PEI coated epoxy modified quartz plate after manual assembly, and secondary coating of an extra PEI layer leads to an enormous amount of misoriented crystals, as shown in Figure S2. This may be caused by the slight surface property difference between glass and quartz plates. Therefore, dip-coating, another known surface pretreatment method of substrates for zeolite crystal attachment, was performed to modify the surface of quartz plates with PEI solutions. Dip-coating with PEI solution in ethanol with a concentration of 0.425 wt.% resulted into uniformly *a*-oriented silicalite-1 monolayer after manual assembly, as shown in Figure 2a in the main text.

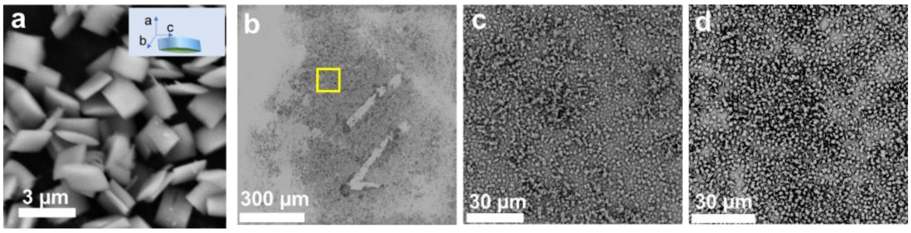


**Figure S2.** a) Scanning electron microscopy (SEM) image of the synthesized *a*-oriented MFI crystals with the order of crystal dimensions $Lc \geq Lb > La$ using trimer-tetrapropylammonium cation (trimer-$TPA^{3+}$) as the structure-directing agent (SDA). b-d) SEM images of b) MFI layers seeded with *a*-oriented slicalite-1 crystals on poly-ethylenimine (PEI) modified quartz substrates using spin-coating. c) Zoom-in SEM image of the highlighted area in b). d) SEM image of the MFI layers seeded with *a*-oriented crystals after two times spin-coating of PEI.

**Summary of the assignment of UV/Vis bands during the methanol-to-hydrocarbons process**

**Table S3.** Assignment of UV/Vis bands during the methanol-to-hydrocarbons process.

| Wavelength (nm) | Assignment | References |
|---|---|---|
| 263-274 | Neutral methylbenzenes | [19] |
| | Monoenyl carbocations | [20] |
| | Polyalkylaromatics | [21] |
| ~ 295 | Polyalkyl-substituted cyclopentadienium ion with four or five alkyl groups | [19] |
| 320-345 | Dienyl carbocations | [20] |
| 353-360 | Up to four methylbenzenium ions | [19] |
| 380-410 | Pentamethylbenzenium and hexamethylbenzenium ions | [22–24] |
| 410-420 | Two and three-ring, neutral polyaromatics | [25–27] |
| | Ethyl-substituted aromatic species | [28] |
| | Methylated naphthalene carbocations | [20,29] |
| 560-635 | Four- and five-ring aromatics | [25] |
| >675 | Polycondensed aromatics | [25] |

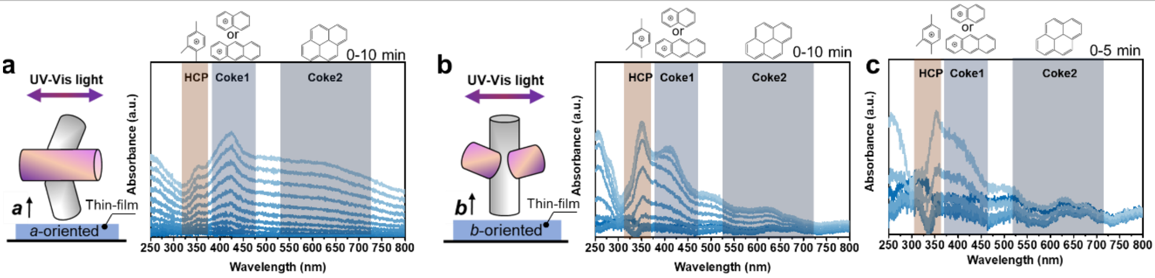


**Figure S3.** *Operando* UV/Vis diffuse reflectance spectroscopic (DRS) data over a) *a*-oriented and b) *b*-oriented zeolite ZSM-5 thin films grown from a secondary growth media with Si/Al= 45 at 623 K during the methanol-to-hydrocarbons (MTH) process for time on stream (TOS)= 0 - 10 min. c) Zoom-in spectra of b) for TOS= 0-5 min. Note that the left insets in a) and b) are schematic of polarization-dependent *operando* UV/Vis DRS over the *a*-oriented and *b*-oriented zeolite ZSM-5 thin films, respectively. c) shows that the absorbance at > 500 nm in the first 5 min of reaction is noisy and oscillating. Therefore, the examination of coking behavior at external surface was primarily focused on the spectra for TOS > 5 min (300 s). The results (Figure S3a) show a much higher relative absorption intensity of internal coke species (coke1) to HCP species for *a*-oriented zeolite ZSM-5 thin films than that for *b*-oriented zeolite ZSM-5 thin films (Figure S3b). Furthermore, much higher intensity ratio of external coke species (coke2) to internal coke species (coke1) was observed at the surface of *a*-oriented ZSM-5 zeolite thin films. The results show that linear/internal polyaromatics (coke 1) can readily form in the straight channels, and the external coke species (coke 2) are more rapidly formed at the surface of the sinusoidal channels.

**DFT calculations of hydrocarbon pool (HCP) species and coke species in the zeolite ZSM-5 channels**

Purely siliceous MFI, *i.e.,* silicalite-1, was applied to study the thermodynamic stability of zeolite-trapped hydrocarbons, as it was demonstrated by Brogaard *et al.* that the thermodynamic stability of hydrocarbon species is primarily determined by their van der Waals interaction with the zeolite framework.[11] Toluene and potential internal coke molecules (naphthalene and anthracene) with different sizes were studied along both the *a*-oriented (sinusoidal) and *b*-oriented (straight) zeolite channels of silicalite-1, shown in Figures S5 and S6. As summarized in Table 1 in the main text, we first examined the adsorption energy differences at the intersections and found small differences (< 5 kJ/mol) in the adsorption energies of toluene and naphthalene, suggesting that the adsorption sites of these molecules are independent of the channel orientations. The anthracene adsorbs more strongly when oriented in the direction of straight channels (about 11.3 kJ/mol) which can be explained in terms of the steric constraints, that is, longer molecules such as anthracene partially extend to channels and therefore are affected by the channel tortuosity, making the adsorption towards sinusoidal channels unfavorable. Overall, the adsorption energies of HCP species at intersection with orientation along the sinusoidal and straight channels, respectively, are between -86.5 and - 99.8 kJ/mol and between -89.7 and -111.1 kJ/mol. In both situations is the adsorption weakest for toluene and strongest for anthracene. However, the increment in the adsorption energy per additional -CH group is much lower when HCP species adsorbed along the sinusoidal channels further supporting an idea that molecules are more sterically constrained.

Further study of the adsorption of these molecules in the zeolite channels showed that all molecules preferred to locate in the straight channels, particularly for anthracene with the largest energy difference of 86.9 kJ/mol compared to the sinusoidal counterpart. It has been generally accepted that the largest molecule that can diffuse out from 10-membered ring zeolites is 1,2,4-trimethylbenzene. Therefore, the coke molecules (naphthalene and anthracene) that are preferentially located in the straight channels will be trapped, consistent with the strong UV/Vis DRS absorption at ~ 420 nm of the hydrocarbons in the corresponding channels.

We have further identified the effect of steric constrains on the stability of the molecules in the zeolite framework. To ensure that the results are independent from the direction of pulling the reverse NEB were performed on the calculated molecules, the extent of confinement imposed on the molecules was expressed via a coordination number. It is computed as a summation over different Si-C, Si-H, O-H and O-C pairs. Figure S7 shows a correlation between the normalized adsorption energy and the average coordination number of the atoms in the studied molecules, that is, the increase of the coordination number leads to less favorable adsorption of the molecules. We find that all molecules have a higher coordination number and lower normalized adsorption energy when positioned inside the sinusoidal channels compared to the straight channels. This can be attributed to the steric structure of the sinusoidal channels, where severe distortion of large molecules, *e.g.,* naphthalene and anthracene, was observed to fit the topology of the sinusoidal channels, inducing extra strain in the planar conformation. These findings are consistent with the results from previous calculations[11] and experiments,[21] that polycyclic arenes prefer to locate along the straight channels of zeolite ZSM-5.

a. Nudged elastic band (NEB) as a method to estimate the thermodynamic stability of molecules within the zeolite ZSM-5 channels

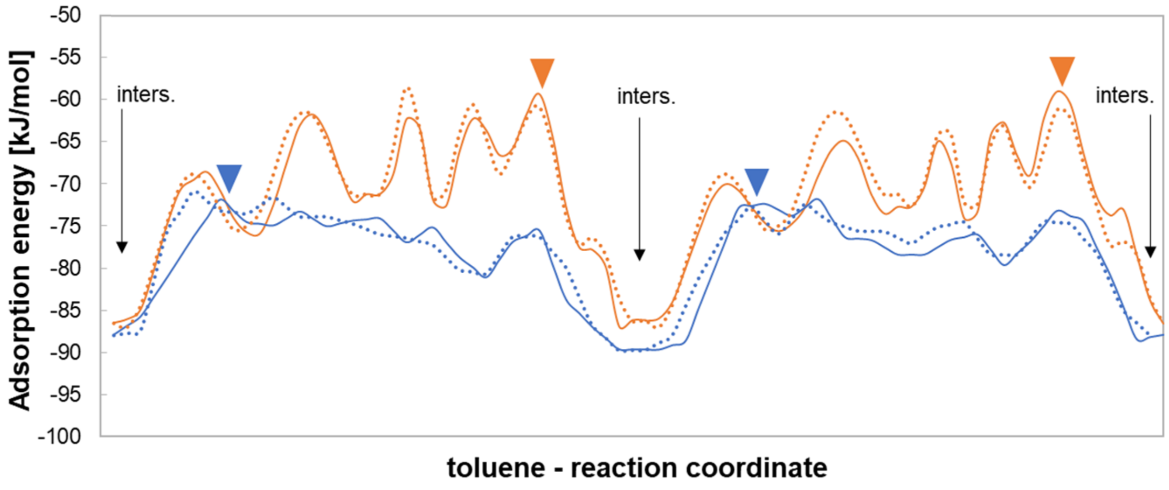


**Figure S4**. To ensure that the minimum energy pathway (MEP) localized using nudged elastic band NEB method is independent from the point direction of the molecule along the same channel orientation we repeated the NEB calculations for toluene in the reverse direction (dashed lines). We find a very good agreement between the localized MEPs with deviations below 5 kJ/mol meaning that the obtained MEPs are independent from the point direction of the studied molecules along the zeolite channels. The MEPs depicted with solid lines were therefore used for further analysis, as shown in Figure S5.

b. Modelling thermodynamic stability of the studied molecules using the Nudged elastic band (NEB) method

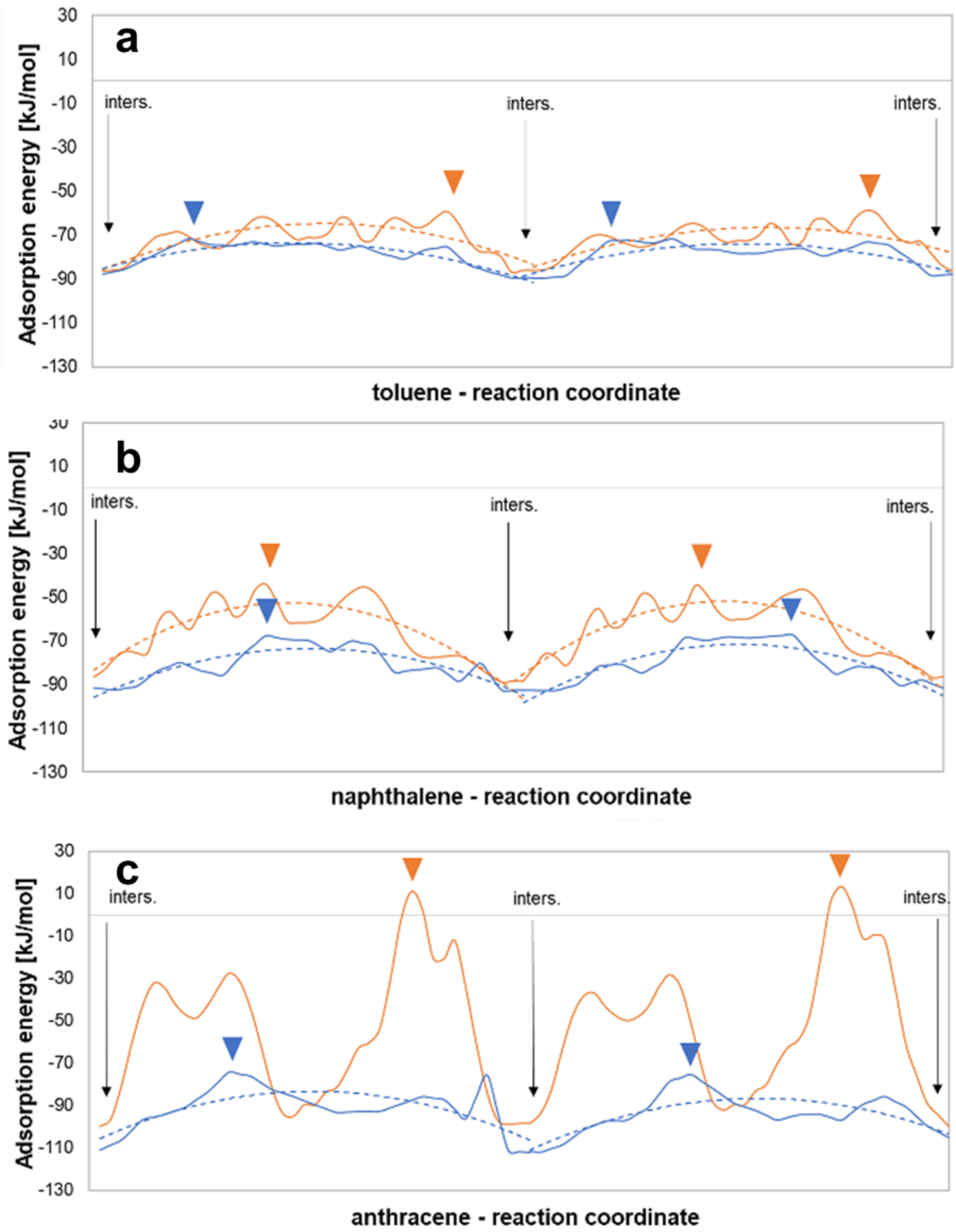


**Figure S5**: Minimum energy pathways (MEP) for a) toluene, b) naphthalene and c) anthracene along the zeolite ZSM-5 channels. MEPs indicate how difficult it would be for arenes to diffuse towards either sinusoidal (orange line) or straight channels (blue line) from a thermodynamic point of view. The energy difference between the least stable points on MEPs along sinusoidal and straight channels increases with increasing molecule size, suggesting the preference of the HCP species to be located along the straight channels. To visually capture these differences, the potential energy profiles between intersections were approximated with parabolic fitting. MEPs also shows that several local minima exist along the channel.

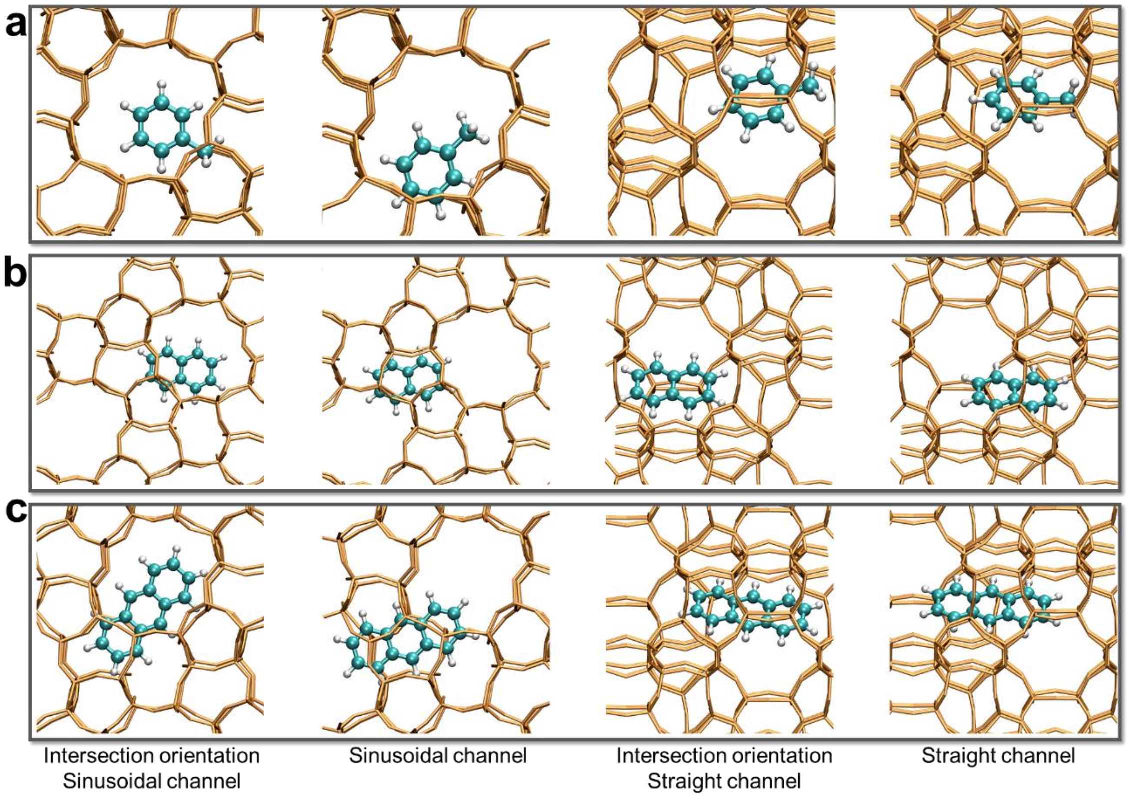


**Figure S6.** Visualization of the most and least stable structures on minimum energy paths (MEPs) for a) toluene, b) naphthalene and c) anthracene located along different positions of the zeolite channels. The most stable structures are always located at the channel intersection, while the least stable structures are located either in the middle of the sinusoidal or straight channels or at the entrance to the channels (as in the case of toluene when oriented along the straight channels). This is because we explored the potential energy profiles along the whole channels and the least stable position does not necessarily have to be in the middle of the channel, but it is rather a result of confinement and a strain induced by the channel on the molecule.

c. The influence of zeolite confinement on the thermodynamic stability of the studied molecules

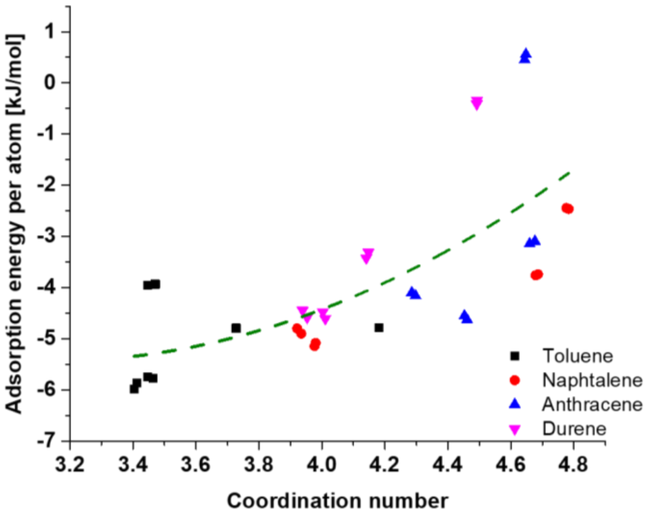


**Figure S7.** The correlation between the normalized adsorption energy and the average (normalized) coordination number. All stationary points of minimum energy paths (MEPs) along both channel orientations were analyzed and the number of intermolecular contacts between the framework atoms (Si) and studied molecules was counted. Note that the coordination number was computed based on the number of carbon and hydrogen atoms surrounded by framework atoms. Higher coordination number means more steric constraints is imposed on the molecule from the framework. The results show that all molecules have a higher coordination number and lower normalized adsorption energy when positioned inside the sinusoidal channels compared to the straight channels. Moreover, it has been found that the increase of the coordination number leads to less favorable adsorption of the molecules. To further explore the effect of confinement, the results for durene have also been added.

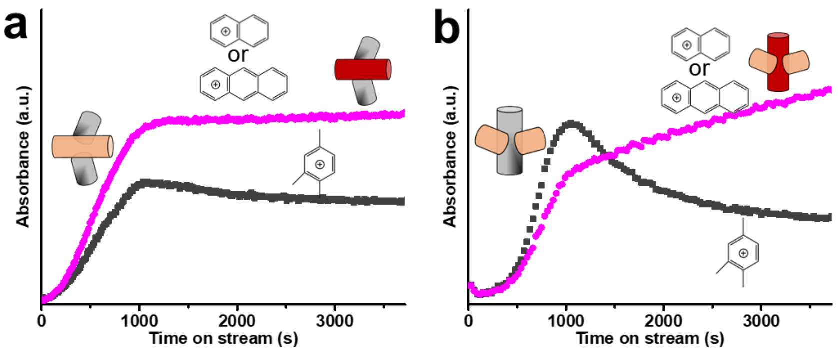


**Figure S8.** Time on stream (TOS) development of hydrocarbon pool (HCP) species (dark grey) at ~ 360 nm and linear polyaromatic species (pink) at ~ 420 nm over a) an *a*-oriented and b) a *b*-oriented thin films during the methanol-to-hydrocarbons (MTH) process at 623 K. a) shows that linear polyaromatic species inside the straight channels accumulated faster, demonstrating that the straight channels can be readily blocked. This also led to the deficiency of methanol in b) the *b*-oriented zeolite thin films. Therefore, the remaining HCP species continuously consumed into products and aromatic species, resulting in the decrease and disequilibrium of HCP species after ~ 1000 s. Note that orange and red colors represent initially blocked and fully blocked channels, respectively.

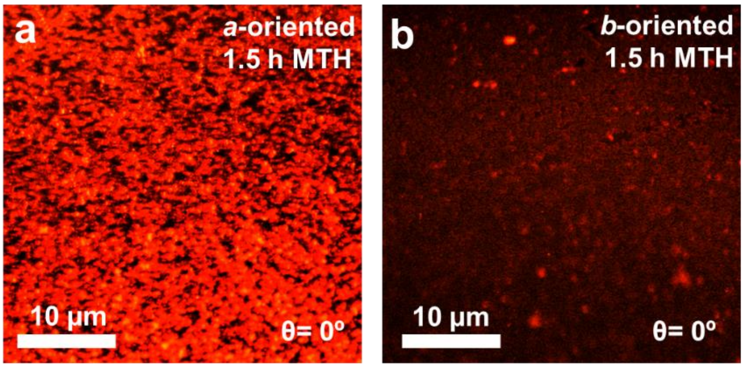


**Figure S9.** Confocal fluorescence micro-spectroscopic images of external coke species (excitation= 561 nm, emission > 580 nm) in a) an *a*-oriented thin film and b) a *b*-oriented thin film at their horizontal positions after 1.5 h the methanol-to-hydrocarbons (MTH) process. The results show that external polyaromatics were readily formed at the surface of the sinusoidal channels.

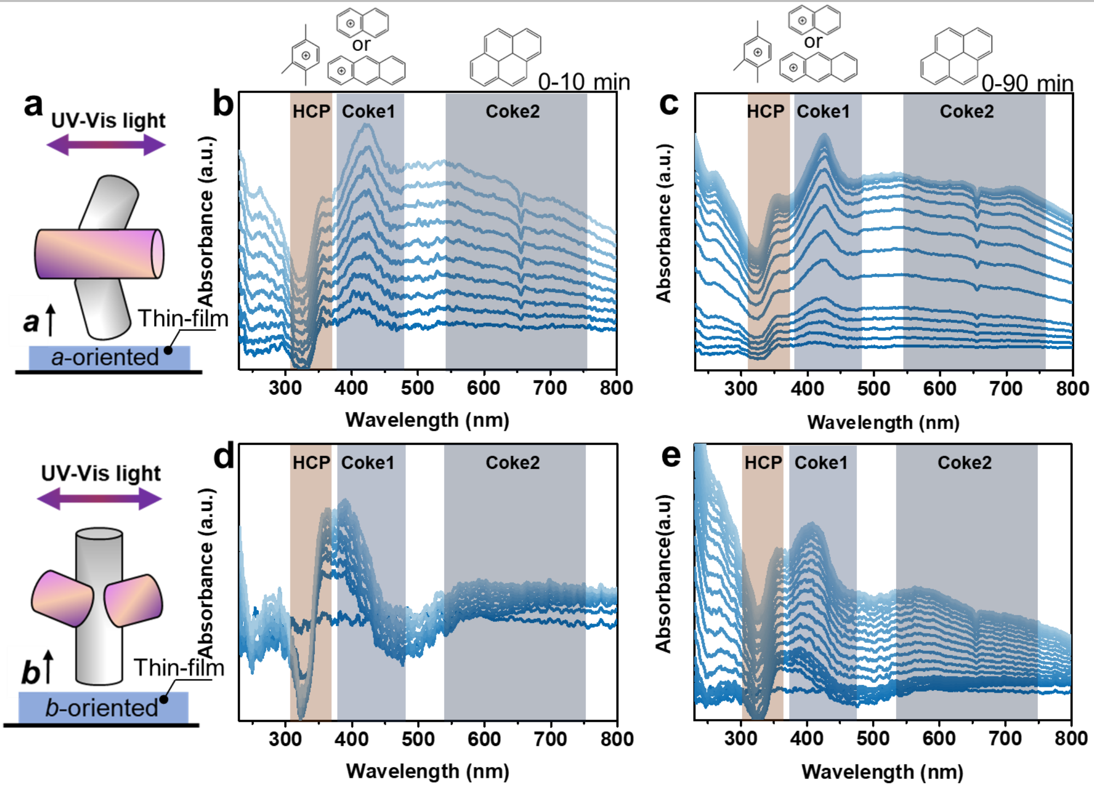


**Figure S10.** a) Schematic of polarization-dependent *operando* UV/Vis diffuse reflectance spectroscopy (DRS) during the methanol-to-hydrocarbons (MTH) process over *a*-oriented (top) and *b*-oriented (bottom) zeolite ZSM-5 thin films. b,d) *Operando* UV/Vis DRS data over the b) *a*-oriented and d) *b*-oriented zeolite ZSM-5 thin films grown from a secondary growth media (SGM) solution with Si/Al= 125 during the MTH process at 623 K for time on stream (TOS)= 0 - 10 min. c,e) *Operando* UV/Vis DRS data over the c) *a*-oriented and e) *b*-oriented zeolite ZSM-5 thin films grown from a SGM solution with Si/Al= 125 during the MTH process at 623 K for TOS= 0 - 90 min. The results (Figure S10b) show a much higher relative absorption intensity of internal coke species (coke1) to HCP species for *a*-oriented zeolite ZSM-5 thin films than that for *b*-oriented zeolite ZSM-5 thin films (Figure S10). Furthermore, much higher intensity ratio of external coke species (coke2) to internal coke species (coke1) was observed at the surface of *a*-oriented ZSM-5 zeolite thin films. This further corroborates that linear/internal polyaromatics (coke 1) can readily form in the straight channels, and the external coke species (coke 2) are more rapidly formed at the surface of sinusoidal channels.

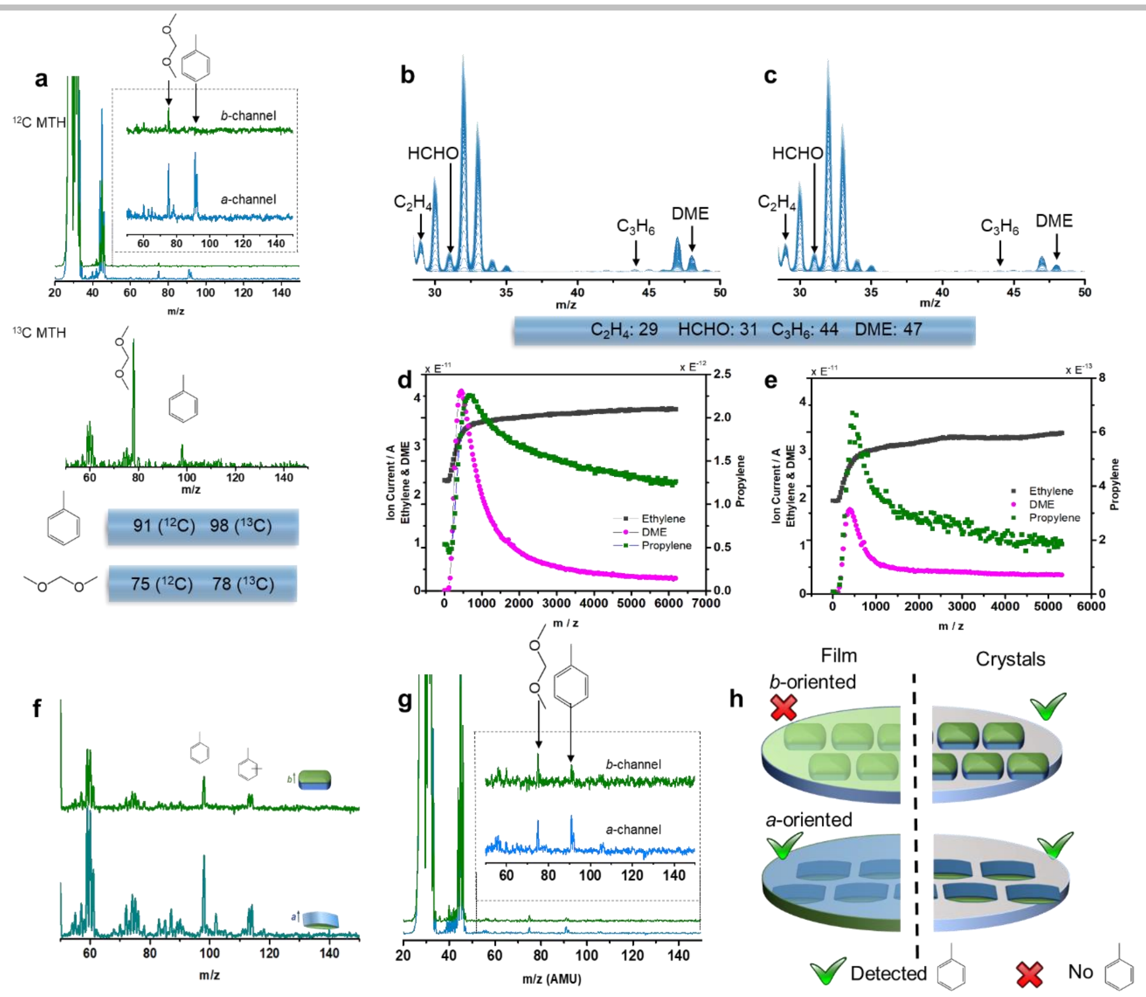


**Figure S11.** a) Representative mass spectrometry (MS) results of the products effluent of (top) $^{12}$C MTH process at 1200s time on stream (TOS) over *a*-oriented (blue) and *b*-oriented thin films (green) grown from a secondary growth media (SGM) solution with Si/Al= 45 and (bottom) $^{13}$C labeled MTH process over the *a*-oriented thin films with Si/Al= 45 at 623 K. b,c) 2-D plot of MS results of the products effluent from b) *a*-oriented and c) *b*-oriented thin films during $^{13}$C labeled methanol-to-hydrocarbons (MTH) process at 623 K. d,e) Time dependent MS results of ethylene (29), propylene (44) and dimethyl ether (47) over the d) *a*-oriented and e) *b*-oriented thin films. f) Representative MS results of $^{13}$C labeled the MTH over *a*-oriented (blue) and *b*-oriented (green) zeolite ZSM-5 crystals with Si/Al= 45 at 1200s time on stream (TOS). g) Representative MS results of $^{12}$C labeled MTH over *a*-oriented (blue) and *b*-oriented (green) zeolite ZSM-5 thin films grown from a SGM solution with Si/Al= 125 at 1200s TOS. h) Schematic of toluene effluent from *a*-oriented (blue) and *b*-oriented (green) zeolite ZSM-5 thin films/crystals. The results revealed the existence of dimethyl ether and C2-C3 olefins, demonstrating that both thin films are indeed active for the MTH process. Moreover, experiments with discrete zeolite ZSM-5 crystals corroborated that toluene indeed preferentially diffused out from the sinusoidal channels.

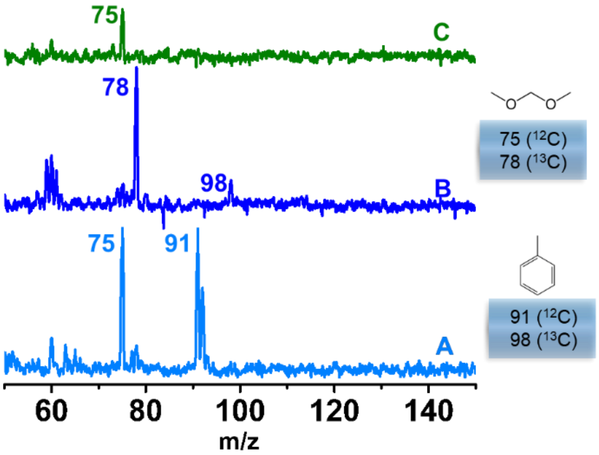


**Figure S12.** Representative mass spectrometry (MS) profiles of the products effluent from (A and B) *a*-oriented and (C) *b*-oriented zeolite ZSM-5 thin films grown in a secondary growth media solution with Si/Al= 45 during the methanol-to-hydrocarbons (MTH) process at 623 K (as shown in Figure S11). Note that traces (A) and (B) are the MS profiles from the $^{12}C$ and $^{13}C$ labeled MTH process, respectively. The data confirms that toluene is in the effluent products from the sinusoidal channels with m/z= 91 ($^{12}C$ methanol) and 98 ($^{13}C$ methanol).

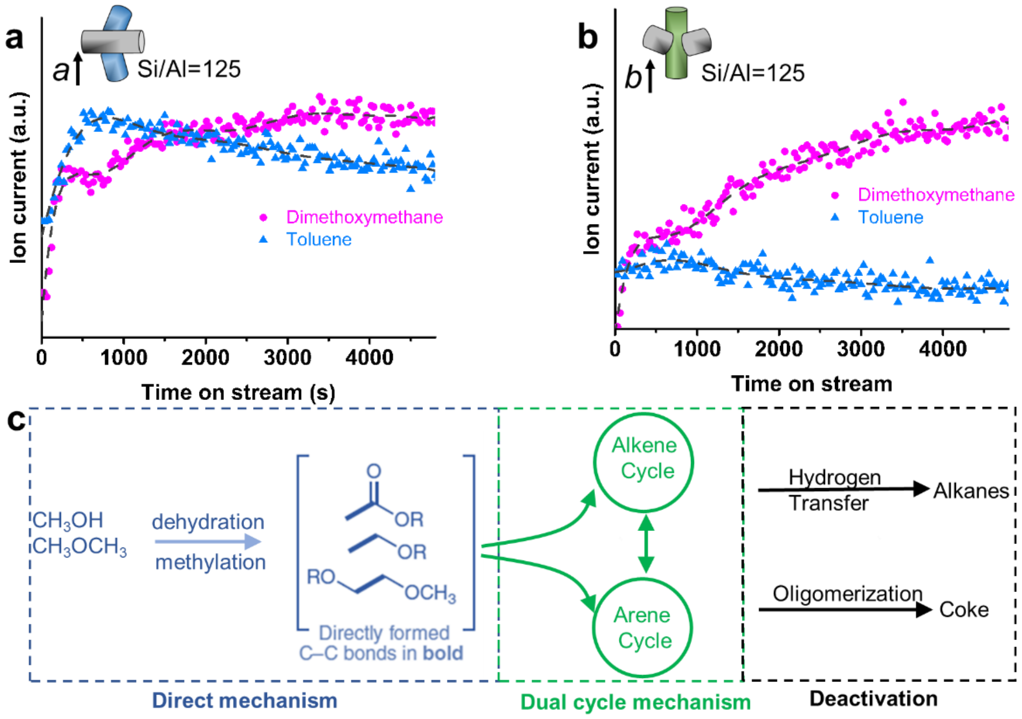


**Figure S13.** a-b) Mass spectrometry (MS) profiles of dimethoxymethane (DMM) and toluene over a) *a*-oriented and b) *b*-oriented zeolite ZSM-5 thin films grown from a secondary growth media (SGM) solution with Si/Al= 125. c) The simplified scheme of the reaction mechanism of the methanol-to-hydrocarbons (MTH) process reveals that hydrocarbon pool (HCP) species (*e.g.,* toluene and xylene) formed after the direct C-C species (acetic acid and dimethoxymethane, R= $CH_3$).[30] Moreover, the aromatic/arene cycle not only leads to the formation of light olefins, but also it supplies coke precursors and is responsible for the deactivation. Dash lines in a) and b) are interpolations to guide the eyes. The MS results show that DMM was detected earlier than toluene, similar to the results obtained from the zeolite ZSM-5 thin films grown from a SGM solution with Si/Al= 45 (Figure 3c). These results directly confirmed the reaction mechanism of the MTH process, *i.e.,* toluene should be produced after DMM that is related to the first C-C bond formation.

***In-situ* UV/Vis diffuse reflectance spectroscopic studies on oligomerization of 4-methoxystyrene and thiophene**

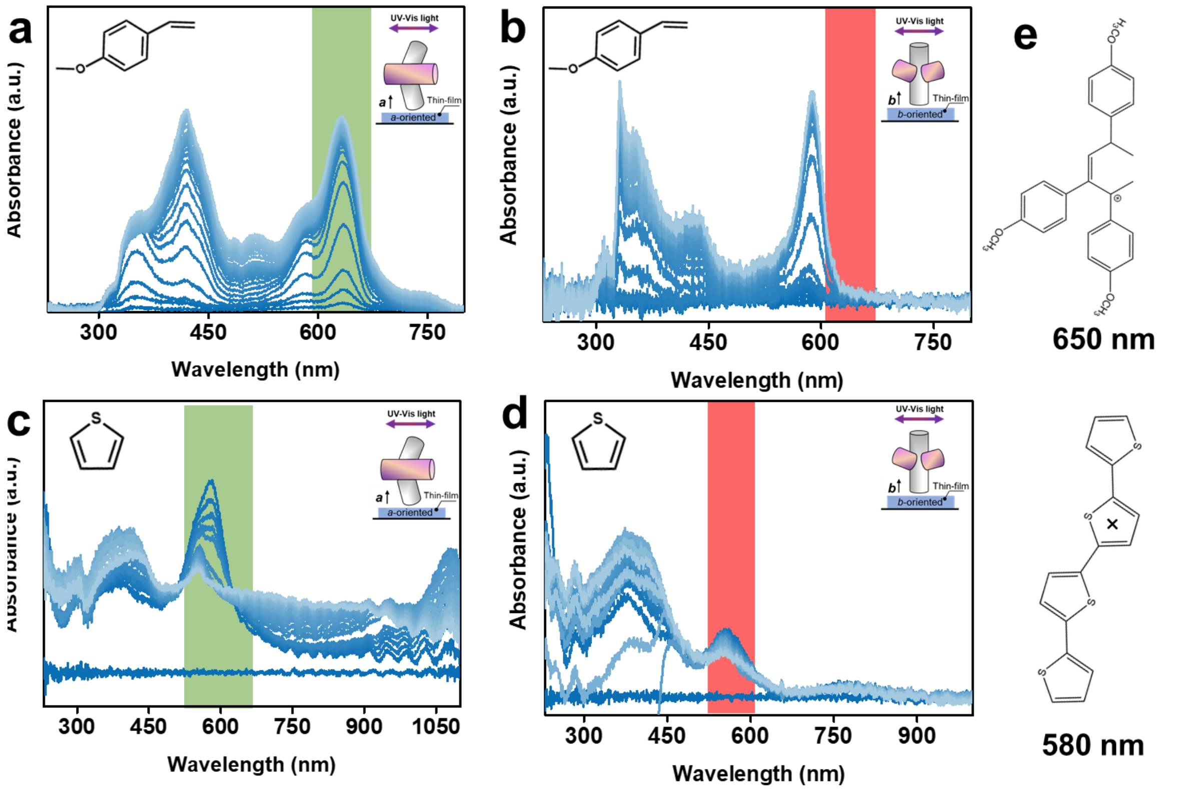


**Figure S14.** a,b) *In-situ* UV/Vis diffuse reflectance spectroscopy (DRS) data of oligomerization of 4-methoxystyrene over a) *a*-oriented and d) *b*-oriented zeolite ZSM-5 thin films grown from a secondary growth media (SGM) solution with Si/Al= 45, respectively. c,d) *In-situ* UV/Vis DRS data of oligomerization of thiophene over c) *a*-oriented and d) *b*-oriented zeolite ZSM-5 thin films grown from a SGM solution with Si/Al= 45, respectively. e) Molecule structures and corresponding adsorption band positions of large oligomers from 4-methoxystyrene (top) and thiophene (bottom). Note the highlighted absorption band range shows the formation (green) or absence (red) of extended molecules. The results show that more extended molecules are readily formed in the straight channels for both oligomerization reactions.

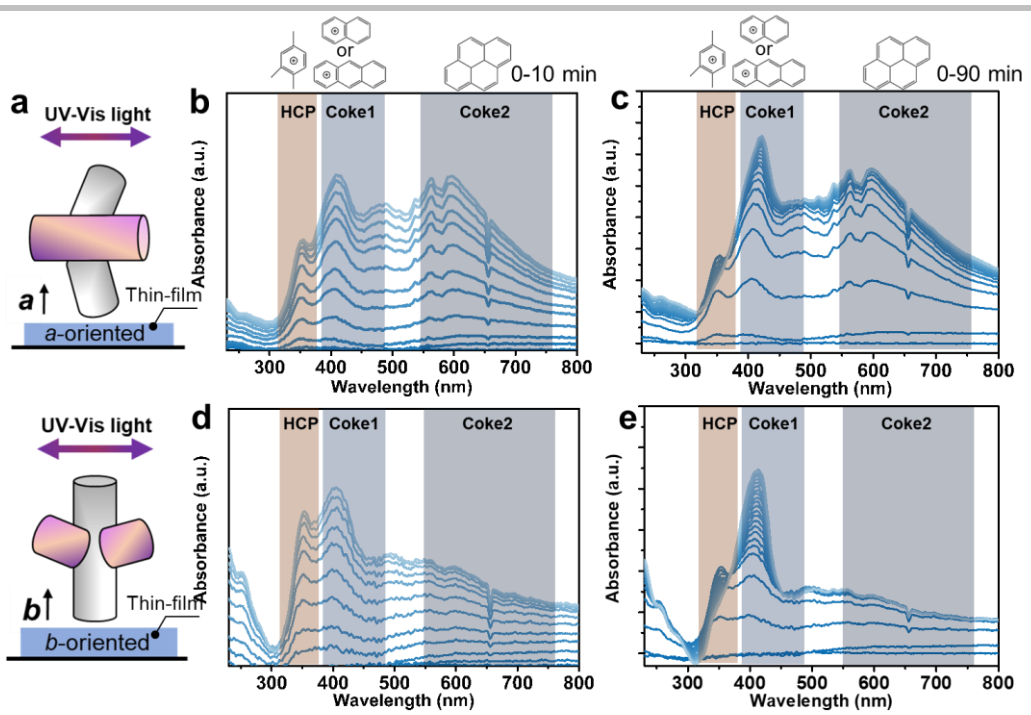


**Figure S15.** a) Schematic of polarization-dependent *operando* UV/Vis diffuse reflectance spectroscopy (DRS) during the ethanol-to-hydrocarbons (ETH) process over *a*-oriented (top) and *b*-oriented (bottom) zeolite ZSM-5 thin films. b,d) *Operando* UV/Vis DRS data over the b) *a*-oriented and d) *b*-oriented zeolite ZSM-5 thin films grown from a secondary growth media (SGM) solution with Si/Al= 45 during the ETH process at 623 K for time on stream (TOS)= 0 - 10 min. c,e) *Operando* UV/Vis DRS data over the c) *a*-oriented and e) *b*-oriented zeolite ZSM-5 thin films grown from a SGM solution with Si/Al= 45 during the ETH process at 623 K for TOS= 0 - 90 min. The results (Figure S15b) show a much higher relative absorption intensity of internal coke species (coke1) to HCP species for *a*-oriented zeolite ZSM-5 thin films than that for *b*-oriented zeolite ZSM-5 thin films (Figure S15d). Furthermore, much higher intensity ratio of external coke species (coke2) to internal coke species (coke1) was observed at the surface of *a*-oriented ZSM-5 zeolite thin films. This suggests that linear/internal polyaromatic coke species (coke 1) can readily form in the straight channels, and the external coke species (coke 2) are more prompt at the surface of sinusoidal channels during the ETH process, being consistent with the results obtained from the MTH process.

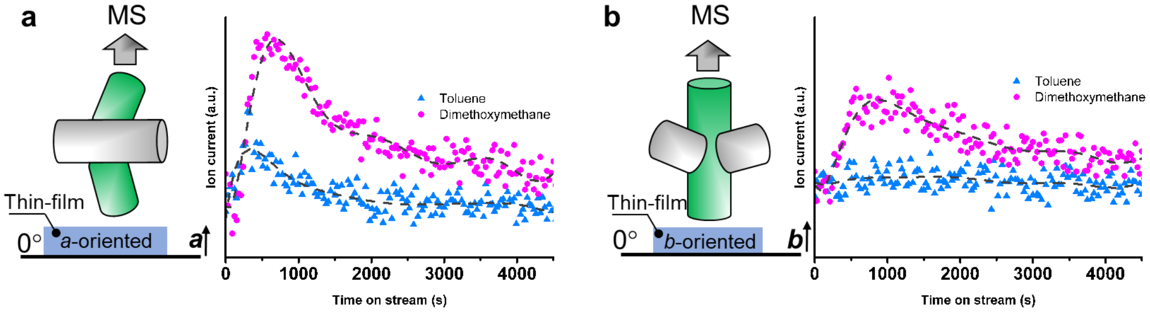


**Figure S16.** Mass spectrometry (MS) profiles of dimethoxymethane (DMM) and toluene over a) an *a*-oriented and b) a *b*-oriented thin films grown from a secondary growth media solution with Si/Al= 125 formation during the ethanol-to-hydrocarbons (ETH) process at 623 K. Dash lines are interpolations to guide the eye. The results show that toluene was in the effluent products from the sinusoidal channels, demonstrated by the increase of ion current of toluene from 0 to ~ 500 s. Conversely, no toluene was observed in the effluent products from the straight channels, being consistent with the results obtained from the methanol-to-hydrocarbons (MTH) process. Moreover, the maximum ion current of DMM was detected earlier than that of toluene during the MTH process, while it was detected after toluene during the ETH process. It has been demonstrated in the MTH process that DMM is related to the first C-C formation (Figure S13c). However, the first C-C bond formation is unnecessary in the ETH process, as it involves a $C_2$ reactant.[31] Thus, these experiments also suggest that the zeolite thin films can be used as model systems to study catalytic chemical reaction mechanisms.

**Physicochemical properties of anisotropic crystals**

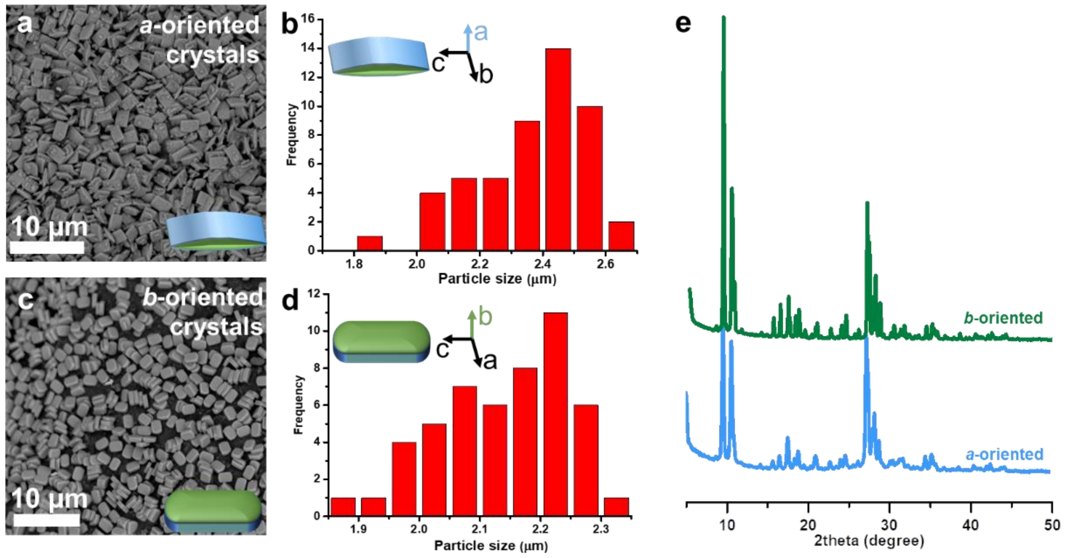


**Figure S17.** a-d) Scanning electron microscopy (SEM) images and particle size distributions of anisotropic zeolite ZSM-5 crystals. a,c) SEM images of a) *a*-oriented and c) *b*-oriented zeolite ZSM-5 crystals synthesized in the solution with Si/Al= 125; b,d) Particle size distribution in *c*-axis of b) *a*-oriented and d) *b*-oriented zeolite ZSM-5 crystals. e) X-ray diffraction (XRD) patterns of the *a*-oriented (blue) and *b*-oriented (green) zeolite ZSM-5 crystals in a) and c), respectively. Note that the SEM images are the same as shown in Figures 5a and 5b. The insets in b) and d) are schematic illustrations of the crystallographic orientations of *a*-oriented and *b*-oriented zeolite ZSM-5 crystals, respectively. The results demonstrate that the crystals are well crystallized with comparable crystal sizes and acidity.

**Table S4.** Physicochemical properties of anisotropic crystals.

| Sample | Crystal size (μm) | | | $NH_3$-TPD (μmol/g) |
|---|---|---|---|---|
| | *a*-axis (μm) | *b*-axis (μm) | *c*-axis (μm) | |
| *a*-oriented ZSM-5 | 0.55 ±0.07 | 1.42 ±0.11 | 2.36 ±0.18 | 172 |
| *b*-oriented ZSM-5 | 1.43 ±0.08 | 0.66 ±0.05 | 2.13 ±0.10 | 145 |

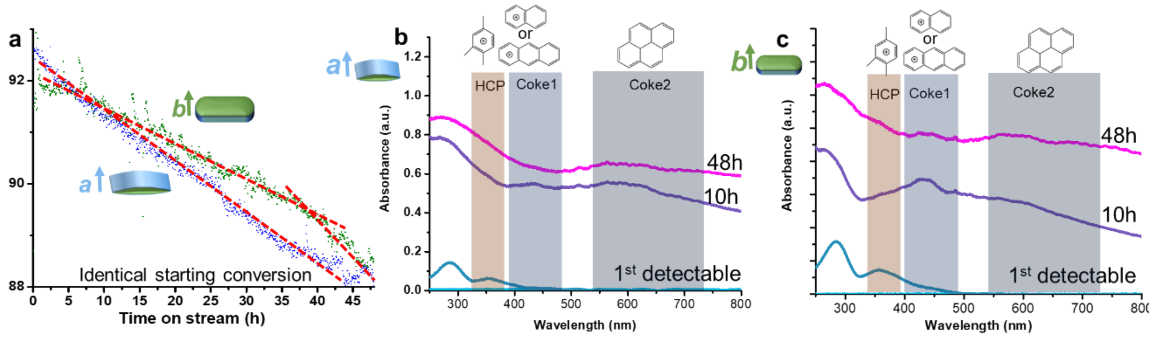


**Figure S18.** a) Deactivation rates measured at the same starting methanol conversion with variable weight hourly space velocity (WHSV) over the *a*-oriented (blue) and *b*-oriented (green) zeolite ZSM-5 crystals grown from a growth media solutions with Si/Al= 45 during 48 h of the methanol-to-hydrocarbons (MTH) process at 623 K. b,c) *Operando* UV/Vis diffuse reflectance spectroscopy (DRS) data of the b) *a*-oriented and c) *b*-oriented zeolite ZSM-5 crystals grown from the growth media solutions with Si/Al= 45 during 48 h of the MTH process at 623 K with an identical WHSV of 5 $h^{-1}$ ( spectra profile colors corresponding to those of the arrows in Figure 5d) .

**Table S5.** Absorbance intensity ratio of external coke species (~ 640 nm) to internal coke species (~ 420 nm) for *a*-oriented and *b*-oriented crystals during the methanol-to-hydrocarbons process at 623 K.

| Reaction time (h) | Intensity ratio of 640 nm to 420 nm (a.u.) | |
|---|---|---|
| | ***a*-oriented crystals** | ***b*-oriented crystals** |
| 10 | 1.0 | 0.81 |
| 48 | 1.0 | 0.99 |

**Characterization of the retained hydrocarbons**

The total weight and properties of coke were analyzed using thermogravimetric analysis coupled with mass spectrometry (TGA-MS).[29,32] The TGA-MS results summarized in Figure S19a show more coke species on *a*-oriented zeolite crystals than *b*-oriented zeolite crystals, being consistent with the faster deactivation of the *a*-oriented zeolite crystals. Interestingly, an additional peak at 1020 K was observed in the MS profile of the *b*-oriented crystals, as shown in Figure S19b. It has been reported that the porosity of the coked catalysts may determine the apparent activation energy for combustion of coke inside the pores.[33] Specifically, the heavily blocked pores will drastically increase the diffusion limitation of oxygen, resulting in ultra-low concentration of oxygen in these pores compared to that of the bulk carrier gas.[32] Therefore, these coke species will be consumed in ultra-high (> 973 K) temperatures after the complete combustion of external coke species. Thus, we tentatively assign the shoulder peak at 1020 K in Figure S19b to internal coke species. These results corroborate the previous conclusion that the internal coke species are prompt to be formed in and block straight channels.

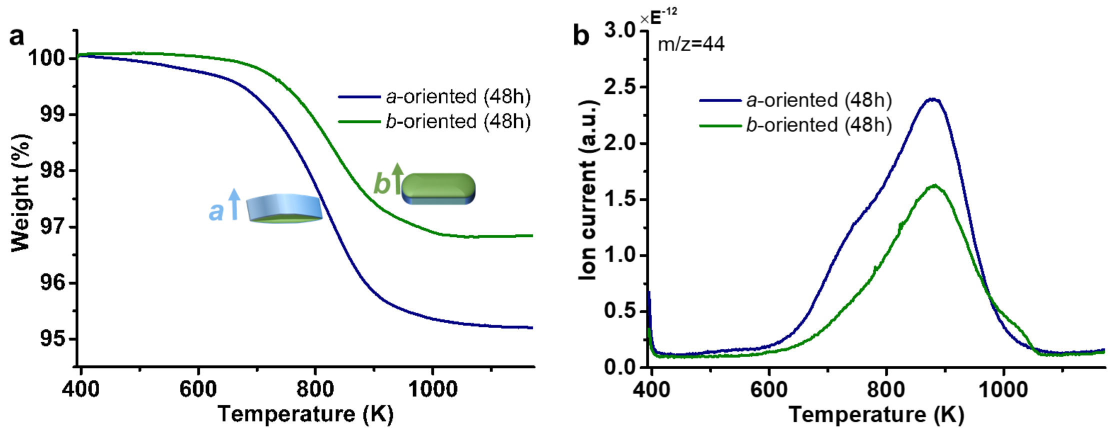


**Figure S19.** a) Thermogravimetric analysis (TGA) of coked *a*-oriented (blue) and *b*-oriented zeolite ZSM-5 crystals after 48 h the methanol-to-hydrocarbons (MTH) reaction. b) The corresponding on-line mass spectrometry (MS) profiles of $CO_2$ (m/z= 44) as a function of temperature.

## References


[1] J. Choi, S. Ghosh, L. King, M. Tsapatsis, *Adsorption* **2006**, *12*, 339–360.
[2] T. C. T. Pham, H. S. Kim, K. B. Yoon, *Science* **2011**, *334*, 1533–1538.
[3] J. Choi, S. Ghosh, Z. Lai, M. Tsapatsis, *Angew. Chem. Int. Ed.* **2006**, *45*, 1154–1158.
[4] J. P. Perdew, K. Burke, M. Ernzerhof, *Phys. Rev. Lett.* **1996**, *77*, 3865–3868.
[5] J. Hutter, M. Iannuzzi, F. Schiffmann, J. VandeVondele, *Interdiscip. Rev. Comput. Sci*. **2014**, *4*, 15–25.
[6] S. Grimme, *J. Comp. Chem.* **2006**, 27, 1787-1799**.**
[7] F. Göltl, A. Grüneis, T. Bučko, J. Hafner, *J. Chem. Phys.* **2012**, *137*, 114111.
[8] F. Göltl, J. Hafner, *Micropor. Mesopor. Mat.* **2013**, *166*, 176–184.
[9] S. Goedecker, M. Teter, J. Hutter, *Phys. Rev. B* **1996**, *54*, 1703–1710.
[10] J. VandeVondele, J. Hutter, *J. Chem. Phys.* **2007**, *127*, 114105.
[11] R. Y. Brogaard, B. M. Weckhuysen, J. K. Nørskov, *J. Catal.* **2013**, *300*, 235–241.
[12] M. Boronat, A. Corma, *ACS Catal.* **2019**, *9*, 1539–1548.
[13] G. Henkelman, B. P. Uberuaga, H. Jónsson, *J. Chem. Phys.* **2000**, *113*, 9901–9904.
[14] M. Iannuzzi, A. Laio, M. Parrinello, *Phys. Rev. Lett.* **2003**, *90*, 238302.
[15] S. S. Batsanov, *Inorg. Mat.* **2001**, *37*, 871–885.
[16] I. Yarulina, K. D. Wispelaere, S. Bailleul, J. Goetze, M. Radersma, E. Abou-Hamad, I. Vollmer, M. Goesten, B. Mezari, E. J. M. Hensen, J. S. Martínez-Espín, M. Morten, S. Mitchell, J. Perez-Ramirez, U. Olsbye, B. M. Weckhuysen, V.V. Speybroeck, F. Kaptein, J. Gascon, *Nat. Chem.* **2018**, *10*, 804–812.
[17] G. T. Whiting, N. Nikolopoulos, I. Nikolopoulos, A. D. Chowdhury, B. M. Weckhuysen, *Nat. Chem.* **2018**, *11*, 23–31.
[18] E. Kim, J. Choi, M. Tsapatsis, *Micropor. Mesopor. Mat.* **2013**, *170*, 1–8.
[19] M. J. Wulfers, F. C. Jentoft, *ACS Catal.* **2014**, *4*, 3521–3532.
[20] E. Borodina, H. Sharbini Harun Kamaluddin, F. Meirer, M. Mokhtar, A. M. Asiri, S. A. Al-Thabaiti, S. N. Basahel, J. Ruiz-Martinez, B. M. Weckhuysen, *ACS Catal.* **2017**, *7*, 5268–5281.
[21] W. Dai, X. Wang, G. Wu, L. Li, N. Guan, M. Hunger, *ChemCatChem* **2012**, *4*, 1428–1435.
[22] T. Liang, J. Chen, Z. Qin, J. Li, P. Wang, S. Wang, G. Wang, M. Dong, W. Fan, J. Wang, *ACS Catalysis* **2016**, *6*, 7311–7325.
[23] M. Bjørgen, F. Bonino, S. Kolboe, K.-P. Lillerud, A. Zecchina, S. Bordiga, *J. Am. Chem. Soc.* **2003**, *125*, 15863–15868.
[24] L. Palumbo, F. Bonino, P. Beato, M. Bjørgen, A. Zecchina, S. Bordiga, *J. Phys. Chem. C* **2008**, *112*, 9710–9716.
[25] D. Mores, J. Kornatowski, U. Olsbye, B. M. Weckhuysen, *Chem. Eur. J.* **2011**, *17*, 2874–2884.
[26] V. Van Speybroeck, K. Hemelsoet, K. De Wispelaere, Q. Qian, J. Van der Mynsbrugge, B. De Sterck, B. M. Weckhuysen, M. Waroquier, *ChemCatChem* **2013**, *5*, 173–184.
[27] K. Hemelsoet, Q. Qian, T. De Meyer, K. De Wispelaere, B. De Sterck, B. M. Weckhuysen, M. Waroquier, V. Van Speybroeck, *Chem. Eur. J.* **2013**, *19*, 16595–16606.
[28] F. L. Bleken, K. Barbera, F. Bonino, U. Olsbye, K. P. Lillerud, S. Bordiga, P. Beato, T. V. W. Janssens, S. Svelle, *J. Catal.* **2013**, *307*, 62–73.
[29] J. Goetze, F. Meirer, I. Yarulina, J. Gascon, F. Kapteijn, J. Ruiz-Martínez, B. M. Weckhuysen, *ACS Catal.* **2017**, 4033–4046.
[30] I. Yarulina, A. D. Chowdhury, F. Meirer, B. M. Weckhuysen, J. Gascon, *Nature Catalysis* **2018**, *1*, 398–411.
[31] A. D. Chowdhury, A. Lucini Paioni, G. T. Whiting, D. Fu, M. Baldus, B. M. Weckhuysen, *Angew. Chem. Int. Ed.* **2019**, 3908–3912.
[32] X. Xian, C. Ran, C. Nai, P. Yang, S. Zhao, L. Dong, *Appl. Catal. A: Gen.* **2017**, *547*, 37–51.
[33] C. Le Minh, R. A. Jones, I. E. Craven, T. C. Brown, *Energy Fuels* **1997**, *11*, 463–469.
